# Insulin Resistance Is Not Conserved in Myotubes Established from Women with PCOS

**DOI:** 10.1371/journal.pone.0014469

**Published:** 2010-12-30

**Authors:** Mette Eriksen, Ann Dorte Pørneki, Vibe Skov, Jorge S. Burns, Henning Beck-Nielsen, Dorte Glintborg, Michael Gaster

**Affiliations:** 1 Department of Endocrinology, Odense University Hospital, Odense, Denmark; 2 University of Southern Denmark, Odense, Denmark; 3 Clinic for Molecular Endocrinology, Department of Endocrinology, Odense University Hospital and Medical Biotechnology Center, University of Southern Denmark, Odense, Denmark; 4 Department of Clinical Biochemistry and Pharmacology and Department of Clinical Genetics, Human MicroArray Center, Odense University Hospital, Odense, Denmark; Louisiana State University, United States of America

## Abstract

**Background:**

Polycystic ovary syndrome (PCOS) is the most common endocrine disorder among premenopausal women, who often develop insulin resistance. We tested the hypothesis that insulin resistance in skeletal muscle of patients with polycystic ovary syndrome (PCOS) is an intrinsic defect, by investigating the metabolic characteristics and gene expression of in vitro differentiated myotubes established from well characterized PCOS subjects.

**Methods:**

Using radiotracer techniques, RT-PCR and enzyme kinetic analysis we examined myotubes established from PCOS subjects with or without pioglitazone treatment, versus healthy control subjects who had been extensively metabolically characterized in vivo. Results Myotubes established from PCOS and matched control subjects comprehensively expressed all insulin-sensitive biomarkers; glucose uptake and oxidation, glycogen synthesis and lipid uptake. There were no significant differences between groups either at baseline or during acute insulin stimulation, although in vivo skeletal muscle was insulin resistant. In particular, we found no evidence for defects in insulin-stimulated glycogen synthase activity between groups. Myotubes established from PCOS patients with or without pioglitazone treatment also showed no significant differences between groups, neither at baseline nor during acute insulin stimulation, although in vivo pioglitazone treatment significantly improved insulin sensitivity. Consistently, the myotube cultures failed to show differences in mRNA levels of genes previously demonstrated to differ in PCOS patients with or without pioglitazone treatment (PLEK, SLC22A16, and TTBK).

**Conclusion:**

These results suggest that the mechanisms governing insulin resistance in skeletal muscle of PCOS patients in vivo are not primary, but rather adaptive.

**Trial Registration:**

ClinicalTrials.gov NCT00145340

## Introduction

Polycystic ovary syndrome (PCOS) is the most common endocrine disorder among premenopausal women affecting 5–8% of women of reproductive age [1]–[2]. PCOS is characterized by hyperandrogenism, chronic anovulation and/or polycystic ovaries [3]–[4]. More than 50% of PCOS patients are insulin resistant and have a diabetes risk 5–8 times higher than age- and weight-matched controls [5]. The exact mechanism for insulin resistance in PCOS is, however, still unknown [6]. Skeletal muscle is the major site of insulin mediated glucose disposal (Rd) and muscular insulin resistance is a major risk factor for type 2 diabetes (T2D) [7] in PCOS [8]. It is debated whether insulin resistance in PCOS is similar to insulin resistance in T2D, or if it represents an exclusive subphenotype [9]–[11]. The insulin receptor affinity and number in PCOS patients is equivalent to that of controls, insulin resistance is presumably mediated through downstream changes in the insulin receptor-mediated signal transduction cascade [12]. Metabolic studies of PCOS patients have shown that impaired insulin-stimulated glucose metabolism is largely accounted for by reduced non-oxidative glucose metabolism (NOGD) [9]. In agreement with these results, impaired insulin-stimulated glycogen synthesis has been documented *in vivo* in skeletal muscle from women with PCOS [13]. Furthermore, impaired insulin signaling through AKT and AS160 changes associated with hyperandrogenemia has been documented in skeletal muscle cells from women with PCOS [10].

The genetic contribution leading to PCOS is not completely clarified, and the pathophysiological complexity of the syndrome complicates genetic analyses [14]. Kashar-Miller *et al.* found a PCOS prevalence of 35% and 40% in premenopausal mothers and sisters of PCOS patients [15]. PCOS is considered an oligogenetic syndrome and several candidate genes involved in insulin action, glucose and lipid metabolism have been proposed. [16]–[17]. Alterations at the translational level have also been demonstrated, including down regulation of genes involved in mitochondrial oxidative phosphorylation, associated with impaired insulin-stimulated total, oxidative and nonoxidative glucose disposal in PCOS patients [18]. A recent microarray study by Skov *et al.* [19] demonstrated significant differential expression of several genes in the skeletal muscle of women with PCOS after treatment with pioglitazone.

Cultured human myotubes display the morphological, metabolic and biochemical properties of adult skeletal muscle and offer a unique model to distinguish between genetic and environmental factors in the etiology of insulin resistance and T2D [20], [21]. We and other have reported a number of potential intrinsic defects in myotubes established from patients with T2D including impaired insulin-stimulated glycogen synthesis [20]; reduced glycogen synthase activity [20]–[22]; reduced basal glucose uptake [20]; impaired glucose oxidation [23], a reduced TCA flux [24]–[25] and a lower basal palmitate oxidation [26] [27]–[28].

Previous *in vitro* studies on whether inborn defects, as seen in myotubes established from T2D subjects, exist in myotubes established from subjects with PCOS found both increased and reduced glucose uptake but without insulin resistance [29]–[30].

To identify primary changes causing impaired insulin responsiveness in PCOS myotubes, we compared the metabolic characteristics of *in vitro* cultured myotubes from PCOS patients and healthy matched controls under normal glycemic and normal insulinemic conditions and after acute insulin stimulation. Extending previous studies, we aimed to investigate if the metabolic improvements detected *in vivo* after 16 weeks pioglitazone treatment were also detectable *ex vivo* in subcultured myotubes in order to clarify whether *in vivo* induced changes by pioglitazone were intrinsically retained in subcultured myotubes.

## Results

### Glucose and lipid metabolism

As reported previously [9], PCOS subjects had increased fasting levels of serum insulin, free testosterone, and plasma triglycerides compared to controls (Table 1). Insulin-stimulated glucose disposal (Rd) was 50% lower in PCOS subjects than controls (*P*<0.001), and this was primarily accounted for by a 60% reduction in NOGM, but also a 39% decrease in glucose oxidation. Treatment of PCOS subjects with pioglitazone significantly reduced fasting serum insulin and improved insulin-stimulated Rd, glucose oxidation, and NOGM. No significant changes were measured in fasting Rd and basal glucose metabolism (Rd, glucose oxidation and NOGM) between PCOS subjects and controls or during pioglitazone treatment (data not shown).

**Table 1:** Clinical and metabolic characteristics of PCOS subjects and control subjects.

Clinical and metabolic characteristics	Control subjects	PCOS patients	PCOS patients pre-treatment	PCOS patients post-treatment
	(n = 14)	(n = 28)	(n = 9)	(n = 9)
**Age (years)**	33.8 (±2.1)	31.6 (±1.2)	29.4 (±2.1)	29.4 (±2.1)
**Weight (kg)**	98.2 (±3.8)	94.9 (±2.1)	95.3 (±2.8)	94.4 (±3.3)
**BMI (kg/m^2^)**	33.7 (±1.7)	33.2 (±0.8)	32.6 (±0.9)	32.5 (±1.5)
**Body fat (%)**	40.5 (±1.6)	40.4 (±1.0)	38.8 (±1.4)	39.5 (±1.5)
**Fasting:**				
**Triglycerides (mmol/l)**	0.86 (±0.11)	1.66 (±0.18)*	1.61 (±0.27	1.15 (±0.18)
**Free testosterone (mg/l)**	0.025 (±0.003)	0.040 (±0.005)*	0.057 (±0.009)	0.048 (±0.007)
**Glucose (mmol/l)**	5.6 (±0.1)	5.9 (±0.1)	5.5 (±0.2)	5.2 (±0.1)
**Insulin (pmol/l)**	51 (±6)	90 (±10)**	113 (±21)	55 (±10)†
**FFA (mmol/l)**	0.47 (±0.04)	0.44 (±0.03)	0.48 (±0.05)	0.53 (±0.06)
**Clamp:**				
**Rd (mg/min/m^2^)**	297 (±23)	150 (±8)*	128 (±17)	172 (±20)†
**Glucose oxidation (mg/min/m^2^)**	141 (±17)	84 (±4)*	80 (±11)	95 (±12)†
**Lipid oxidation (mg/min/m^2^)**	1 (±6)	23 (±2)*	24 (±5)	19 (±5)
**NOGD (mg/min/m^2^)**	157 (±22)	66 (±6)*	48 (±7)	77 (±10)†

Data represents mean ± SEM and P<0.05 is considered significant.

*P≤0.001 vs. control subjects.

**P≤0.01 vs. control subjects.

† P≤0.05 vs. pre-treatment.

### Metabolic pathways in PCOS during in vitro studies

We established subcultures from twenty-eight of thirty PCOS subjects and fourteen healthy control subjects. Glucose transport, glucose oxidation, glycogen synthesis, and lipid uptake were significantly stimulated during acute insulin stimulation in myotubes established from subjects with PCOS and healthy controls. There were no significant differences between groups either at baseline or during acute insulin concentration (1µmol/l) (Table 2). The best described defect in the pathophysiology of T2D is reduced insulin stimulated glycogen synthase activity [31], [32], confirmed in myotubes established from subjects with T2D [20], [22]. Insulin stimulated glycogen synthase activity was also impaired in skeletal muscle of PCOS subjects (Table 1) [13]. We measured glycogen synthase activity in myotubes established from PCOS subjects and controls and found no significant differences at baseline or during acute insulin stimulation (Table 2). In order to test whether the impairments *in vivo* is only translated into myotubes established from subjects with the highest degree of hyperandrogenism or hyperinsulinemia, the PCOS group was subdivided into two groups, individuals with the fourteen lowest (range 0.77–1.89 nmol/l) and fourteen highest (range 1.91–4.24 nmol/l) total testosterone levels respectively, or fourteen lowest (range 38.0–73.0 pmol/l) and fourteen highest (range 79.7–236.3 pmol/l) insulin levels respectively and further tested for differences in metabolic phenotype. Subdividing the PCOS group did not reveal significant differences in the metabolic phenotype between myotubes established from PCOS subjects with hyperandrogenism or hyperinsulinemia compared to low hormone concentrations (Data not shown)

**Table 2:** Metabolic characteristics and gene expression of relevant genes of isolated and cultures skeletal muscle cells from untreated women with PCOS and controls.

Metabolic characteristics	PCOS subjects	Control subjects
	(n = 28)	(n = 14)
**Glucose transport (nmol/min/mg)**		
Baseline	1.99 (±0.15)	1.80 (±0.16)
Acute insulin stimulation	2.24 (±0.17)*	2.16 (±0.21)*
Insulin effect (ratio)	1.16 (±0.04)	1.20 (±0.05)
**Glycogen synthesis (nmol/min/mg)**		
Baseline	0.41 (±0.03)	0.47 (±0.04)
Acute insulin stimulation	0.63 (±0.04)*	0.76 (±0.07)**
Insulin effect (ratio)	1.59 (±0.05)	1.67 (±0.11)
**Glucose oxidation (nmol/min/mg)**		
Baseline	0.15 (±0.01)	0.15 (±0.01)
Acute insulin stimulation	0.20 (±0.02)*	0.18 (±0.02)*
Insulin effect (ratio)	1.34 (±0.08)	1.20 (±0.06)
**Lipid uptake (nmol/min/mg)**		
Baseline	0.84 (±0.02)	0.88 (±0.03)
Acute insulin stimulation	0.96 (±0.03)**	1.04 (±0.04)**
Insulin effect (ratio)	1.15 (±0.01)	1.19 (±0.02)
**Lipid oxidation (nmol/min/mg)**		
Baseline	0.02 (±0.00)	0.02 (±0.00)
**Glycogen synthase activity**		
0.1 mmol/l G6P		
Baseline	0.54 (±0.07)	0.54 (±0.09)
Acute insulin stimulation	0.75 (±0.09)*	0.73 (±0.10)*
10 mmol/l G6P		
Baseline	9.99 (±1.06)	9.73 (±1.03)
Acute insulin stimulation	10.40 (±1.29)	9.86 (±1.20)
FV (0.1/10 mmol/l) %		
Baseline	5.41 (±0.74)	5.33 (±0.52)
Acute insulin stimulation	8.29 (±0.86)*	7.77 (±0.76)*

Data represents mean ± SEM and P<0.05 is considered significant.

*P<0.05, baseline vs. acute insulin stimulation.

**P<0.001, baseline vs. acute insulin stimulation.

### Pioglitazone treatment

In the pioglitazone subgroup, we established subcultures from nine subjects treated with pioglitazone and nine subjects treated with placebo. Pioglitazone treatment *in vivo* resulted in increased oxidative and non-oxidative glucose disposal, as previously reported [9], [33], [34]. To test whether treatment with pioglitazone was remembered in subcultured myotubes from subjects with PCOS, we compared the metabolic characteristics of myotubes established from PCOS subjects before and after pioglitazone treatment and further assessed by quantitative real-time PCR, the expression of genes which are not directly associated to the lipid and glucose metabolism; solute carrier family 22 (SLC22A16), pleckstrin (PLEK) and tau tubulin kinase 2 (TTBK2). A previous microarray study of *in vivo* skeletal muscle tissue of the same PCOS subjects before and after pioglitazone treatment [19] found these transcripts significantly differentially expressed; pleckstrin (PLEK) (p = 0.005, FC  = 4.6), solute carrier family 22 (SLC22A16) (p = 0.03, FC  = 4.2), and tau tubulin kinase (TTBK2) (p = 0.00002, FC  = −4.0). Glucose transport, glucose oxidation, glycogen synthesis, and lipid uptake in myotubes isolated from subjects with PCOS before and after pioglitazone treatment were significantly stimulated during acute insulin stimulation. There were no significant differences between myotubes isolated from subjects with PCOS before and after pioglitazone treatment either at baseline or during acute insulin concentration (1µmol/l) (Table 3). Moreover, no significant changes in expression of the three selected genes were observed (Table 3).

**Table 3:** Metabolic characteristics of isolated and cultured skeletal muscle cells from PCOS subjects **before and after pioglitazone treatment.**

Metabolic characteristics and expression of relevant genes	PCOS subjects pre-treatment	PCOS subjects post-treatment
	n = 9	n = 9
**Glucose transport (nmol/min/mg)**		
Baseline	1.79 (±0.13)	1.79 (±0.12)
Acute insulin stimulation	1.94 (±0.14)*	2.14 (±0.16)*
Insulin effect (ratio)	1.09 (±0.04)	1.20 (±0.06)
**Glycogen synthesis (nmol/min/mg)**		
Baseline	0.33 (±0.03)	0.35 (±0.03)
Acute insulin stimulation	0.52 (±0.05)*	0.57 (±0.05)*
Insulin effect (ratio)	1.58 (±0.08)	1.62 (±0.11)
**Glucose oxidation (nmol/min/mg)**		
Baseline	0.13 (±0.01)	0.14 (±0.01)
Acute insulin stimulation	0.18 (±0.01)*	0.16 (±0.01)*
Insulin effect (ratio)	1.44 (±0.12)	1.20 (±0.13)
**Lipid uptake (nmol/min/mg)**		
Baseline	0.83 (±0.05)	0.82 (±0.03)
Acute insulin stimulation	0.97 (±0.05)*	0.96 (±0.04)*
Insulin effect (ratio)	1.18 (±0.02)	1.17 (±0.03)
**Lipid oxidation (nmol/min/mg)**		
Low insulin stimulation	0.015 (±0.001)	0.014 (±0.001)
**Gene expression**		
**SLC22a16**	1.26E -05 (±2.68E -06)	9.01E -06 (±3.81E -06)
**PLEK**	3.91E -05 (±9.23E -06)	2.15E -05 (±8.20E -06)
**TTBK2**	8.32E -04 (±1.16E -04)	1.30E-03 (±6.27E -04)

Subjects received 16 weeks of treatment with 30 mg pioglitazone once daily.

Data represents mean ± SEM and P<0.05 is considered significant.

*P<0.05, baseline vs. acute insulin stimulation.

## Discussion

Cultured human myotubes represent a well-characterized *in vitro* model system of skeletal muscle in which the extracellular environment can be controlled precisely and kept consistent over time [35]. In the present study, we used this model to compare the metabolic characteristics of myotubes established from PCOS subjects and healthy matched controls under normal glycemic and normal insulinemic conditions and after acute insulin stimulation. We aimed to investigate, if the insulin resistance detected *in vivo* in myotubes was also detectable *in vitro*. In contrast to studies of myotubes established from T2D subjects, we could not find significant signs of primary insulin resistance related to glucose or lipid metabolism in myotubes established from PCOS subjects. In order to further ensure that PCOS subjects did not express major primary defects in the glucose metabolism important for insulin resistance, we assessed the glycogen synthase activity in the same cultures. The most well described defect in the pathophysiology of T2D is reduced insulin stimulated glycogen synthase activity [31], [32] and this was demonstrable in myotubes established from subjects with T2D [20], [22]. Glycogen synthase activity in skeletal muscle tissue of the above PCOS subjects was previously shown to be significantly impaired, but the insulin stimulation of glycogen synthase activity in corresponding established myotubes was not reduced (Table 2). Thus, these data indicate that there are no intrinsic (inborn) defects in insulin mediated glucose and lipid metabolism in skeletal muscle of PCOS subjects, and therefore provide good evidence for the assumption that the impaired metabolic characteristics reported *in vivo* are, in large part, acquired defects caused by *in vivo* environmental factors. Few previous studies were performed *ex vivo* in adipocytes and myotubes from PCOS patients. Corbould *et al* found no impairments of neither basal, nor insulin stimulated glycogen synthesis in adipocytes isolated from PCOS subjects [36]. These data were supported by Ciaraldi *et al*, reporting impaired insulin sensitivity but normal rates of maximal insulin-stimulated glucose transport in isolated adipocytes from PCOS patients vs. controls [30]. Corbould *et al* found no significant differences in glycogen synthesis in myotubes from PCOS subjects compared to healthy weight matched controls [37]. In contrast, Ciaraldi *et al* reported that myotubes from PCOS subjects displayed reduced insulin responsiveness for glucose uptake suggesting that skeletal muscle and adipose tissue contribute differently to insulin resistance in PCOS [30]. PCOS is a heterogenic disease and different characteristics of included PCOS patients may have affected results. In the present study, fasting insulin concentrations were 90 pmol/l in contrast to 196 pmol/l in the study by Ciaraldi *et al* [30] whereas total testosterone levels in both studies were 1.9 nmol/l [9], [30]. We tested whether the impairments *in vivo* is only translated into myotubes established from subjects with the highest degree of hyperandrogenism or hyperinsulinemia, and subdivided the PCOS group into low/high testosterone or low/high insulin and tested for differences in metabolic phenotype. Subdividing the PCOS group did not reveal significant differences in the metabolic phenotype between myotubes established from PCOS subjects with hyperandrogenism or hyperinsulinemia compared to low hormone concentrations.

In cultured fibroblasts from PCOS subjects, insulin stimulated glycogen synthesis was significantly decreased [38]. However, fibroblasts are not primary insulin targets, and more consistently, a previous study by Ciaraldi *et al* also showed no defects in insulin stimulated glycogen synthesis in fibroblasts [39]. Another hallmark of T2D, impaired insulin stimulated glucose transport, previously shown to be conserved in cultured myotubes established from T2D subjects [40], was comparable between PCOS subjects and control subjects in our study. In the study by Ciaraldi *et al* absolute glucose uptake was decreased both at baseline insulin stimulation and during acute insulin stimulation in myotubes established from PCOS subjects compared to control subjects. However, insulin sensitivity, as assessed by an insulin dose-response curve, revealed normal insulin responsiveness and further the expression of GLUT4 was comparable between PCOS subjects and controls [30]. Corbould *et al* found an increase in glucose uptake and GLUT1 abundance, and evidence for intrinsic defects in insulin signaling in myotubes established from PCOS, but comparable GLUT4 content. The defects were insufficient to cause insulin resistance in vitro [37]. Taken together, studies by Corbould *et al*, Ciaraldi *et al* and this study, consistently show no primary impairments in insulin sensitivity in myotubes established from patient with PCOS.

The pathogenesis of PCOS may be characterized by a vicious cycle involving insulin resistance, central obesity and hyperandrogenism [6]. Insulin resistance may be the result of changed body composition [41], and induces hyperinsulinemia, subsequently stimulates ovarian and adrenal hormonal production and inhibits SHBG production leading to increased testosterone activity [6]. Insulin sensitizers, such as thiazolidinediones, e.g. pioglitazone, metformin and lifestyle intervention, improve insulin resistance and thereby insulin stimulation of the adrenals and ovaries decreases. Thiazolidinediones stimulate the peroxisome proliferator-activated (PPAR)-γ receptors in the cell nucleus and thereby activate the transcription of genes that affect glucose and lipid metabolism [42], [43]. This leads to decreased peripheral adipocyte lipolysis, decreased free fatty acid (FFA) levels, and decreased visceral fat mass [44]–[46]. Pioglitazone treatment of PCOS subjects *in vivo* showed improved insulin sensitivity, glucose oxidation, NOGD, and lipid oxidation [9]. Studies on muscle biopsies from treated PCOS subjects confirmed an improved glycogen synthase activity, improved insulin signaling through AKT and AS160 [10], and induced changes in the expression of various transcripts involved in mitochondrial biogenesis, insulin signal transduction and glucose and lipid metabolism during pioglitazone treatment [19]. In myotubes established from the pioglitazone treated subgroup, we found no significant differences in the metabolic pathways, nor in the expressional levels of SLC22A16, PLEK and TTBK2, as previously detected *in vivo* in PCOS patients [19] (Table 3). Thus, the *in vivo* induced changes by pioglitazone were not intrinsically retained in cultured satellite cells differentiated to myotubes, thereby strengthening our conclusion that the impaired metabolic characteristics reported *in vivo* are principally, acquired defects.

There are several potential reasons for our discrepancies between insulin resistance detected in skeletal tissue from PCOS subjects and the lack of a correlating phenotype when analysing cultured myotubes. Firstly, this might reflect a predominantly epigenetic mechanism for the insulin resistance *in vivo*. Epigenetic changes in gene expression not due to alterations in the DNA sequence, can still remain mitotically and transgenerationally hereditary. Epigenetic alterations are known to contribute to development of T2D [47], [48], and epigenetic changes have been suggested to contribute to PCOS [49], [50]. However, a pilot study on global DNA methylation of peripheral blood DNA failed to show significant differences between PCOS subjects and healthy controls [51], which is in agreement with our data suggesting no major hereditary defect governing the insulin mediated glucose metabolism.

It is noteworthy, since we have analysed myotube cultures, that a gene whose expression is down-regulated by methylation *in vivo* may become re-expressed, if cells are grown *ex vivo*. Stem cell differentiation is often preceded by cell mitosis and the replication of methylated DNA produces hemimethylated CpG sites which could cause loss of effective gene silencing at those sites if they are not remethylated by endogenous methyltransferase [52]. Given that a principal target molecule of pioglitazone is PPAR-γ, it is also noteworthy that the effect of forced expression of PPAR-γ on stem cell differentiation was context dependent. It sufficed to induce transdifferentiation of cultured predetermined myoblasts into adipocytes, but it could not induce adipogenesis of satellite cells *in vivo* [53]. Failure to fully reproduce the tissue microenvironment *ex vivo* may mean that complementary genetic defects remain covert, only able to influence the insulin response pathway within the *in vivo* context. Systemic hyperandrogenaemia may be producing a systemic oxidative stress that contributes to insulin resistance [54] and studies on cultured rat myotubes exposed to low (within physiological range) and high testosterone concentrations showed that low testosterone exposure increased IRS-1 and Akt phosphorylation, demonstrating the link between a hyperandrogenic and hyperinsulineamic environment [55]. The fact that PCOS can be linked to genomic variants such as the serum antioxidant enzyme paraxonase and variant IGF2 genes [56] emphasizes the likelihood that multifactorial environmental factors contribute to the final insulin resistance phenotype.

In conclusion, we found no evidence that insulin resistance in skeletal muscle of PCOS subjects *in vivo* is of primary origin, but is likely due to acquired defects. Further studies are needed to explain the underlying causes of this acquired insulin resistance in PCOS patients.

## Materials and Methods

The protocol for this trial and supporting CONSORT checklist are available as supporting information; see Checklist S1 and Protocol S1.

### Ethics statement

The study was approved by the local ethics committee (The Scientific Ethical Committee for Vejle and Funen Counties, now referred to as The Scientific Ethical Committee of the Region of Southern Denmark), and by the Danish Medicines Agency and all subjects gave written informed consent.

### Study subjects

The study subjects have previously been described in papers on insulin signaling, insulin sensitivity and insulin activation of glycogen synthase [9], [10], [13], [33].

In brief, thirty reproductively aged Caucasian women with PCOS were included in the study. Criteria for PCOS were irregular periods with cycle length >35 days in combination with total and/or free testosterone above reference interval (upper limits: total testosterone >1.8, free testosterone >0.035 nmol/l) and/or hirsutism**.** Included subjects had elevated fasting insulin levels (>50 pmol/l) and/or were overweight (body mass index (BMI) ≥30 kg/m^2^). Patients with diabetes (fasting plasma glucose ≥7.0 mmol/l), hypertension, elevated liver enzymes, s-prolactin or s-TSH outside reference interval, renal dysfunction, and congestive heart disease were not included in the study. Subjects refrained from oral contraceptive use for at least three months before evaluation, and no patient took medicine known to affect hormonal or metabolic parameters.

Fourteen healthy Caucasian premenopausal women matched to PCOS subjects for BMI and age were studied as controls. All controls had regular menses (period lengths 28–34 days) and did not suffer from hyperandrogenemia or hirsutism.

After initial examination (see description below), 15 subjects were randomized to pioglitazone 30 mg/day (Actos, Takeda, Lilly A/S) or placebo in a double blind fashion. After a treatment period of 16 weeks, subjects were admitted for repeated examinations similar to the initial evaluation program.

Two subjects were excluded from the study: One subject in the placebo group became pregnant and one subject on pioglitazone treatment experienced side effects (dizziness, ankle edema, and anxiety) and was excluded after one week of treatment. All examinations of the study subjects along with the Euglycemic hyperinsulineamic clamp were carried out at The Department of Endocrinology, Odense University Hospital, Odense, Denmark.

### Examinations

Examinations were performed during the follicular phase in patients with oligomenorrhea and in healthy controls. Patients with amenorrhea (period length >3 months) were examined randomly. The evaluation program of included subjects was performed as previously described [9].

### Clinical examination

Ferriman-Gallwey score, blood pressure, WHR, heart and lung stethoscopy, height and weight were determined in all subjects. Waist circumference was determined as the minimum circumference between the iliac crest and lower costae, whereas the hip circumference was determined as the maximum circumference over the gluteal region [9]. Clinical characteristics of the study subject are presented in table 1.

### Euglycemic hyperinsulineamic clamp

The clamp protocol has previously been described [9]. After 120 minutes basal tracer equilibration period, insulin (Actrapid, Novo Nordisk, Bagsvaerd, Denmark) was infused at a rate of 40 mU/m^2^ min. for 180 min and plasma glucose levels were clamped at approximately 5 mmol/l using a variable infusion rate of 20% glucose. 3-^3^H glucose was added to the glucose infusate to maintain constant levels of plasma specific activity during the clamp period. Indirect calorimetry was performed during the last 40 minutes of the basal and insulin infusion periods using a ventilated hood system (Deltatrac 2, SensorMedics, Yorba Linda, CA) and average gas exchanges were used to calculate glucose- and lipid oxidation rates and total energy expenditure.

Muscle biopsies were obtained from the vastus lateralis muscle using a modified Bergström needle with suction under local anaesthesia. Muscle samples were immediately blotted free of blood, fat and connective tissue and frozen in liquid nitrogen within 30 seconds.

### Calculations

Steele's non-steady state formulas were used to calculate rates of total glucose appearance (Ra) and Rd assuming a glucose distribution volume of 200 ml/kg body weight and a pool fraction of 0.65 [9]. NOGD was calculated as the difference between Rd and glucose oxidation determined by indirect calorimetry. Glucose infusion rate (GIR) during the last 20 minutes of the insulin infusion period was used for calculation of whole body insulin sensitivity (Si). Si was calculated as GIR divided by the incremental increase in insulin concentration from the basal to the insulin-stimulated period during the clamp and divided by the mean glucose concentration during the last 40 minutes of the insulin clamp.

### Assays

Serum total testosterone was analyzed using a specific radioimmunoassay (RIA) after extraction as previously described [57]. This method has a close correlation with the determination of testosterone levels using mass spectrometry. Sex hormone binding globulin (SHBG) was analyzed by time-resolved immunoassay using AutoDELFIA commercial kit (Wallac Oy, Turku, Finland). The intra-assay CV for total testosterone was 8.2% and for SHBG it was 5.2%. The inter-assay CV for total testosterone was 13.8% and for SHBG it was 7.5%. Plasma FFA was analyzed by enzymatic colonimetric reactions (Modular P, Roche). Serum levels of insulin, C-peptide and estradiol were analysed by time-resolved fluoroimmunoassay (AutoDELFIA, Wallac Oy, Turku, Finland). Bedside plasma glucose during the euglycaemic hyperinsulinaemic clamps was measured using the glucose oxidase method (Glucose analyser 2, Beckman Instruments). Tritiated glucose specific activity was determined on barium/zinc deproteinised samples as previously described [58].

### Myotube cultures

Dulbecco's modified Eagle's medium (DMEM), heat inactivated fetal calf serum (FCS), penicillin-streptomycin-amphotericin B, and trypsin-EDTA were obtained from Invitrogen (Invitrogen, Scotland, UK). Ultroser G was purchased from Pall Biosepra (Cergy-Saint-Christophe, France). Protein assay kit was purchased from BioRad (Copenhagen, Denmark). Palmitic acid, L-carnitine, and ECM-gel were purchased from Sigma Chemical Co. (St. Louis, USA). Bovine serum albumin (BSA) (essentially fatty acid free) was from Calbiochem (VWR, Roskilde, Denmark). Insulin Actrapid was from Novo Nordisk (Bagsvaerd, Denmark).

Cell cultures were established as previously described [35], [59], [60]. In brief, muscle tissue was minced, washed and dissociated for 60 min. by three treatments with 0.05% trypsin-EDTA. The cells obtained were seeded for up-scaling on ECM-gel coated dishes after 30 minutes of pre-plating. Growth medium contained DMEM supplemented with 2% heat inactivated FCS, 2% Ultroser G, 50 U/ml penicillin, 50 µg/ml streptomycin and 1.25 µg/ml amphotericin B. Cells were sub cultured twice before final seeding. At 75% confluence, the growth medium was replaced by basal medium (DMEM supplemented with 2% heat inactivated FCS, 50 U/ml penicillin, 50 µg/ml streptomycin, 1.25 µg/ml amphotericin B, and supplemented with 25 pmol/l insulin) in order to induce differentiation. The cells were cultured in humidified 5% CO_2_ atmosphere at 37°C, and medium was changed every 2–3 days. Human myotubes established from women with PCOS, control subjects and PCOS subjects before and after pioglitazone treatment were allowed to differentiate under physiological conditions of insulin (25 pmol/l) and glucose (5.5 mmol/l) for eight days. All myotube cultures were used for analysis on day eight after onset of differentiation.

### Methodological considerations

Optimal culture and differentiation conditions have been previously established for human myotubes cultures [35], [59] and current cultures were adequately established and differentiated in accordance with these procedures. To ensure maximal insulin response in all cultures, we used a maximal insulin concentration of 1 µmol/l instead of 100 nmol/l, which is used by other groups [61]. High insulin level may generate a significant signaling through the IGF-1 receptor which could mask the detection of insulin resistance. Henry et al compared the dose responds for insulin and IGF1 on glucose uptake in human myotubes and found similar sensitivity for insulin and IGF1 [61]. The various metabolic pathways in human myotube respond by different magnitudes to insulin stimulation i.e. glycogen synthesis increases up to two fold, glucose oxidation up to 50%, while glucose uptake only increases 20–40% [23], [62], [63]. In the present study we studied several insulin sensitive pathways in order to reduce the possible limitation in evaluation of insulin resistance only at one level as the glucose transport, thereby increasing the chance to detect insulin resistance, if present.

### Substrate oxidation

Glucose and palmitate oxidation was determined by a 96 multi-well tracer technique as previous described [64]. Substrate oxidation was monitored by incubating myotubes with [1-^14^C]-Palmitate (2.0 µCi/ml) in a final concentration of 0.4 mmol/l palmitate and [^14^C(U)]-glucose (2.0 µCi/ml) in a final concentration of 5.0 mmol/l glucose with subsequent capture of liberated ^14^CO_2_ for 4 h at 37°C. Trapped radioactivity was determined with a Microbeta counter (PerkinElmer, Finland).

### Glucose and lipid uptake

Glucose uptake was measured by capturing 2-[1-^14^C]-deoxy-glucose and lipid uptake was measured as the incorporation of [1-^14^C]-Palmitate (2.0 µCi/ml) as previously described [40], [23]. Radioactivity was determined with a Microbeta counter (PerkinElmer, Finland).

### Glycogen synthesis

Glycogen synthesis was measured as previous described in 96 well plates [23]. Radioactivity was measured with a Microbeta counter (PerkinElmer, Finland).

### Glycogen synthase activity

The GS activity was measured as described by Mandarino et al. [65]. Cells were washed three times with ice-cold PBS, and 700 µl GS buffer (50 mmol/l Hepes, 10 mmol/l EDTA, 100 mmol/l NaF, 5 mmol/l DTT, 1 µmol/l leupeptin, 1 µmol/l pepstatin and 200 µmol/l phenylmethylsulfonyl flouride, pH 7.5) was added to each Petri dish. Cells were scraped from the dishes into cryotubes, sonicated 15 seconds twice at 4°C (Sinirep 150, amplitude 14 microns), frozen and stored at -80°C. Aliquots of sonicate were used for determination of GS activity and protein. GS activity was determined at 0.3 mmol/l UDP-glucose, in parallel incubations with: 0, 0.05, 0.1, 1.0 and 10 mmol/l glucose-6-phosphate. GS activity was expressed as nanomoles of UDP-glucose incorporated into glycogen per minute per milligram of total protein. The specific GS activity was calculated as the above GS activity divided by the amount of GS protein determined by western blotting and expressed as nanomoles of UDP-glucose incorporated into glycogen per minute per OD GS protein. FV_0.1_ (fractional velocity) was calculated as the ratio of GS activity determined at 0.1 mmol/l G6P and 10 mmol/l G6P.

### RNA isolation and real time PCR

*TTBK1* is a serine/threonine/tyrosine kinase that is specifically expressed in the brain [66]; SLC22A16 is an organic cation transporter and is widely expressed at low levels in adult tissues [67]; PLEK is the major substrate of protein kinase C in platelets [68].

The expression of SLC22A16, PLEK and TTBK2 was measured using real time PCR. RNA was prepared from the cells using the ABI PRISM 6100 Nucleic Acid PrepStation (Applied Biosystems, Nearum, Denmark) and the Total RNA Isolation Chemistry Kit (cat. no. 4328773, Applied Biosystems, Nearum, Denmark). Briefly, the cells were lysed in the dish and transferred to the RNA purification tray. The lysate was passed through the tray using vacuum, and the RNA bound to the membrane. The RNA was eluted, and the concentration and purity was measured using a NanoDrop spectrophotometer (Thermo Fischer Scientific, Soeborg, Denmark).

RNA was reverse transcribed using the High Capacity cDNA Reverse Transcription Kit (cat. no. 4368814, Applied Biosystems, Nearum, Denmark). Real-time RT-PCR was performed with the StepOnePlus Real-Time PCR System and SYBRGreen PCR Master Mix (both from Applied Biosystems, Nearum, Denmark). Each sample was run in duplicate using 15 ng RNA equivalents per reaction. The expression value of each gene was normalized against the amount of β-actin and calculated by the ΔΔCt method [69]. The following human specific primers were used (DNA Technology A/S, Denmark): PLEK (forward: GCTGGTATCCAACCAGTCTG, reverse: CATTGAGCAGCGATGAAGCA); SLC22A16 (forward: GCCCTCCTGAGTGGAGTGTTAA, reverse: TTTCATTCTCTGACTCCAGTTTTGC); TTBK2 (forward: GCTTGGCTCGACAATTTACC, reverse: CTGACCAACCACAAACTCCA). The quantification of each target gene and β-ACTIN mRNA was performed in separate tubes. Gene expression levels for each target gene were calculated using the comparative Ct method [(1/(2ΔCt) formula, where ΔCt is the difference between Ct target and Ct-reference] after normalization to β-ACTIN mRNA (PerkinElmer's User Bulletin No. 2). Data were analyzed using optical system software version 3.1 (Bio Rad) and Microsoft Excel 2000 to generate relative expression values [70].

### Statistical analysis

Statistical significance was evaluated using Student's t-test and calculated two tailed P values. Paired analysis was performed for comparison of acute and chronic insulin exposure in the same sets of myotubes.

The data in the text, tables, and figures are given as mean +/− SEM. The statistical analysis was performed with INSTAT 2.01 (GraphPad, USA). P<0.05 was considered to be significant.

## Supporting Information

Checklist S1 CONSORT Checklist
(0.24 MB DOC)

Protocol S1 Danish Medicines Agency - Trial Protocol (Danish version)
(0.13 MB DOC)

## References

[1] Knochenhauer ES, Key TJ, Kahsar-Miller M, Waggoner W, Boots LR (1998). Prevalence of the polycystic ovary syndrome in unselected black and white women of the southeastern United States: a prospective study.. J Clin Endocrinol Metab.

[2] Azziz R, Woods KS, Reyna R, Key TJ, Knochenhauer ES (2004). The Prevalence and Features of the Polycystic Ovary Syndrome in an Unselected Population.. J Clin Endocrinol Metab.

[3] (2004). Revised 2003 consensus on diagnostic criteria and long-term health risks related to polycystic ovary syndrome. Fertil Steril.

[4] Ehrmann DA (2005). Polycystic ovary syndrome.. N Engl J Med.

[5] Glintborg D, Henriksen JE, Andersen M, Hagen C, Hangaard J (2004). Prevalence of endocrine diseases and abnormal glucose tolerance tests in 340 Caucasian premenopausal women with hirsutism as the referral diagnosis.. Fertil Steril.

[6] Glintborg D, Andersen M (2010). An update on the pathogenesis, inflammation, and metabolism in hirsutism and polycystic ovary syndrome.. Gynecol Endocrinol.

[7] Shulman GI (2000). Cellular mechanisms of insulin resistance.. J Clin Invest.

[8] Teede H, Deeks A, Moran L (2010). Polycystic ovary syndrome: a complex condition with psychological, reproductive and metabolic manifestations that impacts on health across the lifespan.. BMC Med.

[9] Glintborg D, Hermann AP, Andersen M, Hagen C, Beck-Nielsen H (2006). Effect of pioglitazone on glucose metabolism and luteinizing hormone secretion in women with polycystic ovary syndrome.. Fertil Steril.

[10] Højlund K, Glintborg D, Andersen NR, Birk JB, Treebak JT (2008). Impaired insulin-stimulated phosphorylation of Akt and AS160 in skeletal muscle of women with polycystic ovary syndrome is reversed by pioglitazone treatment.. Diabetes.

[11] Venkatesan AM, Dunaif A, Corbould A (2001). Insulin resistance in polycystic ovary syndrome: progress and paradoxes.. Recent Prog Horm Res.

[12] Dunaif A, Xia J, Book CB, Schenker E, Tang Z (1995). Excessive insulin receptor serine phosphorylation in cultured fibroblasts and in skeletal muscle. A potential mechanism for insulin resistance in the polycystic ovary syndrome.. J Clin Invest.

[13] Glintborg D, Højlund K, Andersen NR, Hansen BF, Beck-Nielsen H (2008). Impaired insulin activation and dephosphorylation of glycogen synthase in skeletal muscle of women with polycystic ovary syndrome is reversed by pioglitazone treatment.. J Clin Endocrinol Metab.

[14] Deligeoroglou E, Kouskouti C, Christopoulos P (2009). The role of genes in the polycystic ovary syndrome: predisposition and mechanisms.. Gynecol Endocrinol.

[15] Kahsar-Miller MD, Nixon C, Boots LR, Go RC, Azziz R (2001). Prevalence of polycystic ovary syndrome (PCOS) in first-degree relatives of patients with PCOS.. Fertil Steril.

[16] Zhuo G, Feng G, Leng J, Yu L, Jiang Y (2010). A 9-bp deletion homoplasmy in women with polycystic ovary syndrome revealed by mitochondrial genome-mutation screen.. Biochem Genet.

[17] Xita N, Georgiou I, Tsatsoulis A (2002). The genetic basis of polycystic ovary syndrome.. Eur J Endocrinol.

[18] Skov V, Glintborg D, Knudsen S, Jensen T, Kruse TA (2007). Reduced expression of nuclear-encoded genes involved in mitochondrial oxidative metabolism in skeletal muscle of insulin-resistant women with polycystic ovary syndrome.. Diabetes.

[19] Skov V, Glintborg D, Knudsen S, Tan Q, Jensen T (2008). Pioglitazone enhances mitochondrial biogenesis and ribosomal protein biosynthesis in skeletal muscle in polycystic ovary syndrome.. PLoS One.

[20] Gaster M, Petersen I, Højlund K, Poulsen P, Beck-Nielsen H (2002). The diabetic phenotype is conserved in myotubes established from diabetic subjects: evidence for primary defects in glucose transport and glycogen synthase activity.. Diabetes.

[21] Henry RR, Ciaraldi TP, Mudaliar S, Abrams L, Nikoulina SE (1996). Acquired defects of glycogen synthase activity in cultured human skeletal muscle cells: influence of high glucose and insulin levels.. Diabetes.

[22] Henry RR, Ciaraldi TP, Abrams-Carter L, Mudaliar S, Park KS (1996). Glycogen synthase activity is reduced in cultured skeletal muscle cells of non-insulin-dependent diabetes mellitus subjects. Biochemical and molecular mechanisms.. J Clin Invest.

[23] Gaster M, Beck-Nielsen H (2004). The reduced insulin-mediated glucose oxidation in skeletal muscle from type 2 diabetic subjects may be of genetic origin–evidence from cultured myotubes.. Biochim Biophys Acta.

[24] Gaster M (2009). Reduced TCA flux in diabetic myotubes: A governing influence on the diabetic phenotype?. Biochem Biophys Res Commun.

[25] Ortenblad N, Mogensen M, Petersen I, Højlund K, Levin K (2005). Reduced insulin-mediated citrate synthase activity in cultured skeletal muscle cells from patients with type 2 diabetes: evidence for an intrinsic oxidative enzyme defect.. Biochim Biophys Acta.

[26] Gaster M, Rustan AC, Aas V, Beck-Nielsen H (2004). Reduced lipid oxidation in skeletal muscle from type 2 diabetic subjects may be of genetic origin: evidence from cultured myotubes.. Diabetes.

[27] Gaster M, Rustan AC, Beck-Nielsen H (2005). Differential utilization of saturated palmitate and unsaturated oleate: evidence from cultured myotubes.. Diabetes.

[28] Wensaas AJ, Rustan AC, Just M, Berge RK, Drevon CA (2009). Fatty acid incubation of myotubes from humans with type 2 diabetes leads to enhanced release of beta-oxidation products because of impaired fatty acid oxidation: effects of tetradecylthioacetic acid and eicosapentaenoic acid.. Diabetes.

[29] Corbould A, Kim YB, Youngren JF, Pender C, Kahn BB (2005). Insulin resistance in the skeletal muscle of women with PCOS involves intrinsic and acquired defects in insulin signaling.. Am J Physiol Endocrinol Metab.

[30] Ciaraldi TP, Aroda V, Mudaliar S, Chang RJ, Henry RR (2009). Polycystic ovary syndrome is associated with tissue-specific differences in insulin resistance.. J Clin Endocrinol Metab.

[31] Damsbo P, Vaag A, Hother-Nielsen O, Beck-Nielsen H (1991). Reduced glycogen synthase activity in skeletal muscle from obese patients with and without type 2 (non-insulin-dependent) diabetes mellitus.. Diabetologia.

[32] Beck-Nielsen H, Groop LC (1994). Metabolic and genetic characterization of prediabetic states. Sequence of events leading to non-insulin-dependent diabetes mellitus.. J Clin Invest.

[33] Glintborg D, Højlund K, Andersen M, Henriksen JE, Beck-Nielsen H (2008). Soluble CD36 and risk markers of insulin resistance and atherosclerosis are elevated in polycystic ovary syndrome and significantly reduced during pioglitazone treatment.. Diabetes Care.

[34] Glintborg D, Frystyk J, Højlund K, Andersen KK, Henriksen JE (2008). Total and high molecular weight (HMW) adiponectin levels and measures of glucose and lipid metabolism following pioglitazone treatment in a randomized placebo-controlled study in polycystic ovary syndrome.. Clin Endocrinol (Oxf).

[35] Gaster M, Kristensen SR, Beck-Nielsen H, Schroder HD (2001). A cellular model system of differentiated human myotubes.. APMIS.

[36] Corbould A, Dunaif A (2007). The adipose cell lineage is not intrinsically insulin resistant in polycystic ovary syndrome.. Metabolism.

[37] Corbould A, Kim YB, Youngren JF, Pender C, Kahn BB (2005). Insulin resistance in the skeletal muscle of women with PCOS involves intrinsic and acquired defects in insulin signaling.. Am J Physiol Endocrinol Metab.

[38] Book CB, Dunaif A (1999). Selective insulin resistance in the polycystic ovary syndrome.. J Clin Endocrinol Metab.

[39] Ciaraldi TP, Morales AJ, Hickmann MG, Odem-Ford R, Yen SSC (1998). Lack of insulin resistance in fibroblasts from subjects with polycystic ovary syndrome.. Metabolism.

[40] Gaster M, Beck-Nielsen H (2006). Triacylglycerol accumulation is not primarily affected in myotubes established from type 2 diabetic subjects.. Biochim Biophys Acta.

[41] Björntorp P (1996). The android woman–a risky condition.. J Intern Med.

[42] Olefsky JM, Saltiel AR (2000). PPAR gamma and the treatment of insulin resistance.. Trends Endocrinol Metab.

[43] Feinstein DL, Spagnolo A, Akar C, Weinberg G, Murphy P (2005). Receptor-independent actions of PPAR thiazolidinedione agonists: is mitochondrial function the key?. Biochem Pharmacol.

[44] Mayerson AB, Hundal RS, Dufour S, Lebon V, Befroy D (2002). The effects of rosiglitazone on insulin sensitivity, lipolysis, and hepatic and skeletal muscle triglyceride content in patients with type 2 diabetes.. Diabetes.

[45] Miyazaki Y, Mahankali A, Matsuda M, Mahankali S, Hardies J (2002). Effect of pioglitazone on abdominal fat distribution and insulin sensitivity in type 2 diabetic patients.. J Clin Endocrinol Metab.

[46] Boden G, Cheung P, Mozzoli M, Fried SK (2003). Effect of thiazolidinediones on glucose and fatty acid metabolism in patients with type 2 diabetes.. Metabolism.

[47] Stoffers DA, Desai BM, DeLeon DD, Simmons RA (2003). Neonatal exendin-4 prevents the development of diabetes in the intrauterine growth retarded rat.. Diabetes.

[48] Park JH, Stoffers DA, Nicholls RD, Simmons RA (2008). Development of type 2 diabetes following intrauterine growth retardation in rats is associated with progressive epigenetic silencing of Pdx1.. J Clin Invest.

[49] Xita N, Tsatsoulis A (2006). Review: fetal programming of polycystic ovary syndrome by androgen excess: evidence from experimental, clinical, and genetic association studies.. J Clin Endocrinol Metab.

[50] Li Z, Huang H (2008). Epigenetic abnormality: a possible mechanism underlying the fetal origin of polycystic ovary syndrome.. Med Hypotheses.

[51] Xu N, Azziz R, Goodarzi MO (2010). Epigenetics in polycystic ovary syndrome: a pilot study of global DNA methylation.. Fertil Steril.

[52] Bird A (2002). DNA methylation patterns and epigenetic memory.. Genes Dev.

[53] Ban A, Yamanouchi K, Matsuwaki T, Nishihara M (2008). In vivo gene transfer of PPAR gamma is insufficient to induce adipogenesis in skeletal muscle.. J Vet Med Sci.

[54] Liu S, Navarro G, Mauvais-Jarvis F (2010). Androgen excess produces systemic oxidative stress and predisposes to beta-cell failure in female mice.. PLoS One.

[55] Allemand MC, Irving BA, Asmann YW, Klaus KA, Tatpati L (2009). Effect of testosterone on insulin stimulated IRS1 Ser phosphorylation in primary rat myotubes–a potential model for PCOS-related insulin resistance.. PLoS One.

[56] San Millan JL, Corton M, Villuendas G, Sancho J, Peral B (2004). Association of the polycystic ovary syndrome with genomic variants related to insulin resistance, type 2 diabetes mellitus, and obesity.. J Clin Endocrinol Metab.

[57] Lykkesfeldt G, Bennett P, Lykkesfeldt AE, Micic S, Moller S (1985). Abnormal androgen and oestrogen metabolism in men with steroid sulphatase deficiency and recessive X-linked ichthyosis.. Clin Endocrinol (Oxf).

[58] Hother-Nielsen O, Henriksen JE, Staehr P, Beck-Nielsen H (1995). Labelled glucose infusate technique in clamp studies. Is precise matching of glucose specific activity important?. Endocrinol Metab.

[59] Gaster M, Beck-Nielsen H, Schroder HD (2001). Proliferation conditions for human satellite cells. The fractional content of satellite cells.. APMIS.

[60] Gaster M, Schroder HD, Handberg A, Beck-Nielsen H (2001). The basal kinetic parameters of glycogen synthase in human myotube cultures are not affected by chronic high insulin exposure.. Biochim Biophys Acta.

[61] Henry RR, Abrams L, Nikoulina S, Ciaraldi TP (1995). Insulin action and glucose metabolism in nondiabetic control and NIDDM subjects. Comparison using human skeletal muscle cell cultures.. Diabetes.

[62] Gaster M (2007). Metabolic flexibility is conserved in diabetic myotubes.. J Lipid Res.

[63] Gaster M (2007). Insulin resistance and the mitochondrial link. Lessons from cultured human myotubes.. Biochim Biophys Acta.

[64] Gaster M (2009). Reduced lipid oxidation in myotubes established from obese and type 2 diabetic subjects.. Biochem Biophys Res Commun.

[65] Mandarino LJ, Wright KS, Verity LS, Nichols J, Bell JM (1987). Effects of insulin infusion on human skeletal muscle pyruvate dehydrogenase, phosphofructokinase, and glycogen synthase. Evidence for their role in oxidative and nonoxidative glucose metabolism.. J Clin Invest.

[66] Lebouvier T, Scales TM, Williamson R, Noble W, Duyckaerts C (2009). The microtubule-associated protein tau is also phosphorylated on tyrosine.. J Alzheimers Dis.

[67] Koepsell H, Lips K, Volk C (2007). Polyspecific organic cation transporters: structure, function, physiological roles, and biopharmaceutical implications.. Pharm Res.

[68] Baig A, Bao X, Haslam RJ (2009). Proteomic identification of pleckstrin-associated proteins in platelets: possible interactions with actin.. Proteomics.

[69] Livak KJ, Schmittgen TD (2001). Analysis of relative gene expression data using real-time quantitative PCR and the 2(-Delta Delta C(T)) Method.. Methods.

[70] Frederiksen CM, Hojlund K, Hansen L, Oakeley EJ, Hemmings B (2008). Transcriptional profiling of myotubes from patients with type 2 diabetes: no evidence for a primary defect in oxidative phosphorylation genes.. Diabetologia.

