# Supplementary material for: Insulin Resistance Is Not Conserved in Myotubes Established from Women with PCOS
Source: PLoS One. 2010 Dec 30;5(12):e14469. doi: 10.1371/journal.pone.0014469 (PMC3012693; doi:10.1371/journal.pone.0014469)
Supplement: Checklist S1 — CONSORT Checklist (0.24 MB DOC) [file pone.0014469.s001.doc]

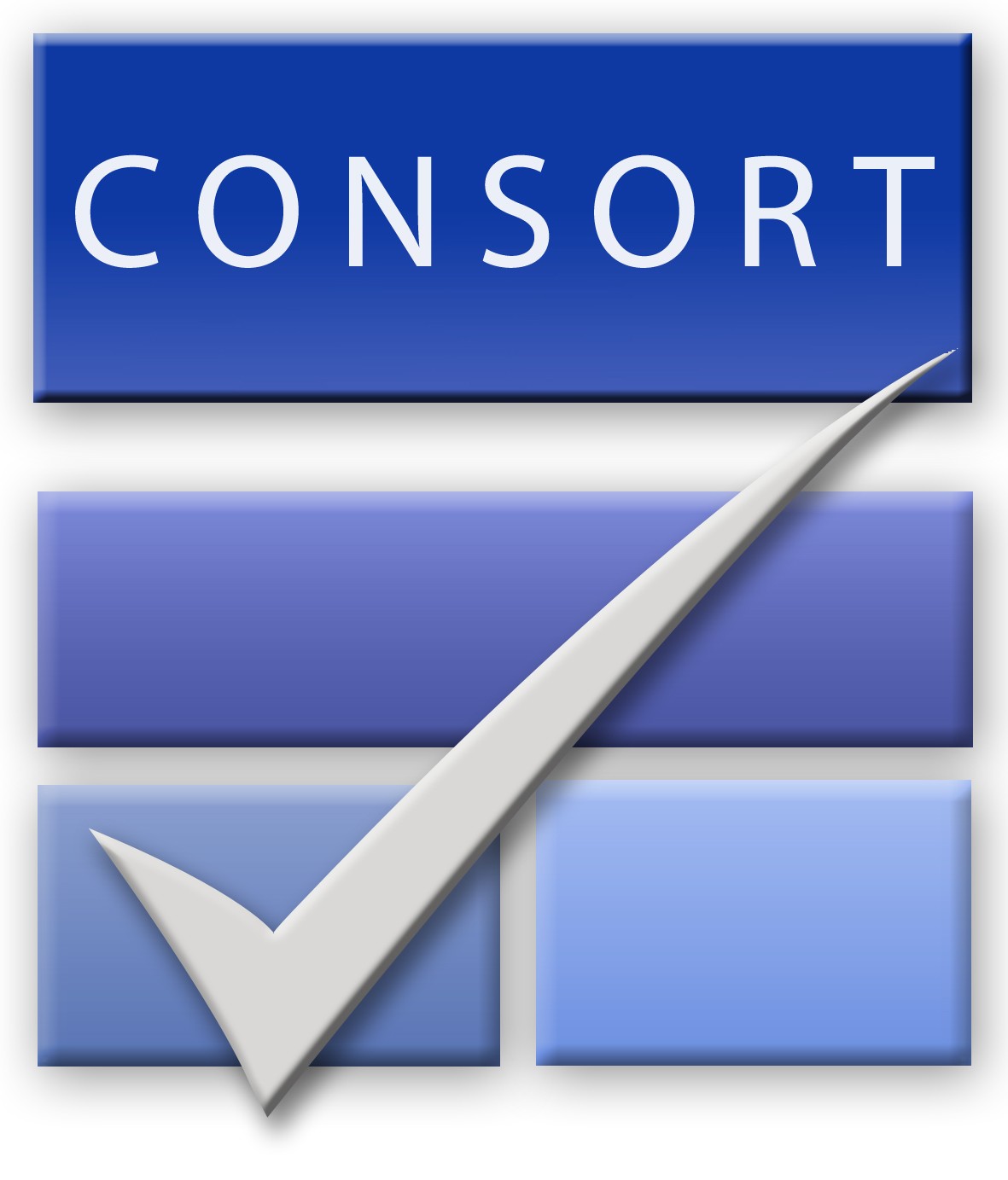
CONSORT 2010 checklist of information to include when reporting a randomised trial*

| Section/Topic | Item No | Checklist item | Reported on page No |
| --- | --- | --- | --- |
| Title and abstract | | | |
|  | 1a | Identification as a randomised trial in the title | No, this study is a spin off study of a previous clinical trial. Including the term “randomised” in the title would, in our view, be misleading to the reader. |
|  | 1b | Structured summary of trial design, methods, results, and conclusions (for specific guidance see CONSORT for abstracts) | This study is a spin off study of a previous clinical trial, and it does not deal with nor discuss any clinical aspects. |
| Introduction | | | |
| Background and objectives | 2a | Scientific background and explanation of rationale | Described in the Introduction section |
|  | 2b | Specific objectives or hypotheses | Described in the Introduction section |
| Methods | | | |
| Trial design | 3a | Description of trial design (such as parallel, factorial) including allocation ratio | Described in the materials and methods sections: study subjects, examinations, clinical examinations, euglycemic hyperinsulineamic clamp, calculations and assays. Additionally: reference is made to CONSORT Checklist section 7a. |
|  | 3b | Important changes to methods after trial commencement (such as eligibility criteria), with reasons | No changes made |
| Participants | 4a | Eligibility criteria for participants | Described in the materials and methods section: study subjects. |
|  | 4b | Settings and locations where the data were collected | Described in the materials and methods section: study subjects. |
| Interventions | 5 | The interventions for each group with sufficient details to allow replication, including how and when they were actually administered | Described in the materials and methods section: study subjects. |
| Outcomes | 6a | Completely defined pre-specified primary and secondary outcome measures, including how and when they were assessed | This study is a spin off study of a previous clinical trial, and it does not deal with nor discuss any clinical aspects. |
|  | 6b | Any changes to trial outcomes after the trial commenced, with reasons | No changes |
| Sample size | 7a | How sample size was determined | This study is a spin off study of a previous clinical trial, and not it self a clinical randomised trial. Throughout the manuscript, reference is made to the previous clinical trial (Glintborg D, Hermann AP, Andersen M, Hagen C, Beck-Nielsen H, Veldhuis JD, Henriksen JE; Fertility and Sterility 86 (2), 2006, 385-397) |
|  | 7b | When applicable, explanation of any interim analyses and stopping guidelines | Reference is made to section 7a |
| Randomisation: |  |  |  |
| Sequence generation | 8a | Method used to generate the random allocation sequence | Reference is made to section 7a. |
|  | 8b | Type of randomisation; details of any restriction (such as blocking and block size) | Described in the materials and methods section: study subjects. |
| Allocation concealment mechanism | 9 | Mechanism used to implement the random allocation sequence (such as sequentially numbered containers), describing any steps taken to conceal the sequence until interventions were assigned | Reference is made to section 7a. |
| Implementation | 10 | Who generated the random allocation sequence, who enrolled participants, and who assigned participants to interventions | Reference is made to section 7a. |
| Blinding | 11a | If done, who was blinded after assignment to interventions (for example, participants, care providers, those assessing outcomes) and how | Described in the materials and methods section: study subjects. |
|  | 11b | If relevant, description of the similarity of interventions | Not relevant. Reference is made to section 7a. |
| Statistical methods | 12a | Statistical methods used to compare groups for primary and secondary outcomes | Materials and methods section: statistical analysis and reference is made to section 7a. |
|  | 12b | Methods for additional analyses, such as subgroup analyses and adjusted analyses | Materials and methods sections: study subjects and examinations. |
| Results | | | |
| Participant flow (a diagram is strongly recommended) | 13a | For each group, the numbers of participants who were randomly assigned, received intended treatment, and were analysed for the primary outcome | Not included in the manuscript, the study is a spin off study of a previous clinical trial. A CONSORT flow diagram is available as supporting information file. Reference is made to section 7a. |
|  | 13b | For each group, losses and exclusions after randomisation, together with reasons | Reference is made to section 13a. |
| Recruitment | 14a | Dates defining the periods of recruitment and follow-up | Reference is made to section 7a and 13a. |
|  | 14b | Why the trial ended or was stopped | Reference is made to section 7a and 13a. |
| Baseline data | 15 | A table showing baseline demographic and clinical characteristics for each group | Table 1 shows the clinical characteristics of the included subjects. Reference is made to section 7a. |
| Numbers analysed | 16 | For each group, number of participants (denominator) included in each analysis and whether the analysis was by original assigned groups | Reference is made to tables 1 and 3. |
| Outcomes and estimation | 17a | For each primary and secondary outcome, results for each group, and the estimated effect size and its precision (such as 95% confidence interval) | Reference is made to section 7a. |
|  | 17b | For binary outcomes, presentation of both absolute and relative effect sizes is recommended | Reference is made to section 7a. |
| Ancillary analyses | 18 | Results of any other analyses performed, including subgroup analyses and adjusted analyses, distinguishing pre-specified from exploratory | Reference is made to section 7a. |
| Harms | 19 | All important harms or unintended effects in each group (for specific guidance see CONSORT for harms) | Materials and methods section: study subjects. |
| Discussion | | | |
| Limitations | 20 | Trial limitations, addressing sources of potential bias, imprecision, and, if relevant, multiplicity of analyses | Not relevant, this is not directly clinical study, but spin off of a clinical study. |
| Generalisability | 21 | Generalisability (external validity, applicability) of the trial findings | Reference is made to section 7a. |
| Interpretation | 22 | Interpretation consistent with results, balancing benefits and harms, and considering other relevant evidence | Not relevant, this is not directly clinical study, but spin off of a clinical study. |
| Other information | | |  |
| Registration | 23 | Registration number and name of trial registry | Materials and methods section: ethics statement, supporting material S1 |
| Protocol | 24 | Where the full trial protocol can be accessed, if available | Materials and methods section: ethics statement, supporting material S1 |
| Funding | 25 | Sources of funding and other support (such as supply of drugs), role of funders | Reference is made to section 7a. |

*We strongly recommend reading this statement in conjunction with the CONSORT 2010 Explanation and Elaboration for important clarifications on all the items. If relevant, we also recommend reading CONSORT extensions for cluster randomised trials, non-inferiority and equivalence trials, non-pharmacological treatments, herbal interventions, and pragmatic trials. Additional extensions are forthcoming: for those and for up to date references relevant to this checklist, see [www.consort-statement.org](http://www.consort-statement.org/).
