# Supplementary material for: Insulin Resistance Is Not Conserved in Myotubes Established from Women with PCOS
Source: PLoS One. 2010 Dec 30;5(12):e14469. doi: 10.1371/journal.pone.0014469 (PMC3012693; doi:10.1371/journal.pone.0014469)
Supplement: Protocol S1 — Danish Medicines Agency - Trial Protocol (Danish version) (0.13 MB DOC) [file pone.0014469.s002.doc]

**Effekten af PPARgamma stimulation**

**på**

**glukosemetabolismen,**

**insulinresistens,**

**væksthormon og cortisol**

**hos kvinder**

**med**

**polycystisk ovariesyndrom.**

Dorte Glintborg, ([dorte.glintborg@dadlnet.dk](mailto:dorte.glintborg@dadlnet.dk)),

Jan Erik Henriksen, ph.d,

Marianne Andersen, ph.d,

Claus Hagen, dr.med,

Henning Beck-Nielsen, professor, dr.med

og

Pernille Hermann, ph.d.

**Endokrinologisk afdeling M, Odense Universitetshospital.**

**Generelle oplysninger:**

**Dato:** 29. august 2026.

**Sponsor:** Endokrinologisk afdeling M, Odense Universitetshospital

**Forsøgsansvarlig investigator:** Administrerende overlæge, dr. Med. Claus Hagen. Endokrinologisk afdeling M, Odense Universitetshospital. 6541 1810.

**GCP:** Forsøget udføres jfr. CGP-reglerne med monitorering foretaget ved GCP-funktionen, afdeling KKA, Klinisk Farmakologi, Odense Universitetshospital, 5000 Odense C.

Forsøget udføres i henhold til nedenstående protokol og i henhold til gældende lovgivning.

**Tidsplan:** Forsøget forventes opstartet juni 2002 og afsluttet februar 2004. Patienter kontaktes med henblik på inklusion og ophør med p-pille behandling juni 2002. Glitazonbehandling forventes opstartet september 2002.

**Praktisk ansvarlig:** Dorte Glintborg under vejledning af: Jan Erik Henriksen, ph.d, Marianne Andersen, ph.d, Claus Hagen, dr.med, Henning Beck-Nielsen, professor, dr.med og Pernille Hermann, ph.d.

**Underskrift:**

Claus Hagen, overlæge, dr. Med

29. august 2026

_______________________________________________________________________________________

**Baggrundsinformation:**

**Præparat:** Tablet Actos a 30 mg. Indeholder pioglitazon.

Registreringsindehaver: Takeda. Repræsenteres i Danmark af Eli Lilly A/S.

Placebotabletter: Fremstilles af Central apoteket ved Odense Universitetshospital. Vedrørende indholdsstoffer henvises til medfølgende blanket.

**Resume af relevante resultater vedrørende glitazonbehandling af patienter med PCOS samt baggrunds litteratur vedrørende forsøget:**

**Introduktion**

7-10 % af kvinder i den reproduktive alder har polycystisk ovariesyndrom (PCOS) (1). Ved PCOS ses forstyrrelser, som indbefatter insulinresistens, hyperinsulinæmi og forhøjet androgenniveau. (1,2) Man kender endnu ikke den udløsende faktor for PCOS (2, 3).

Hyperinsulinæmi stimulerer ovarierne til øget androgenproduktion via enzymet p450c17alfa. (3, 4, 5 ) Øget aktivitet i dette enzym bevirker en øget 17 hydroxylase aktivitet og en øget 17,20 lyase aktivitet. Dette giver sig til udtryk i en øget produktion af 17-hydroxyprogesteron, som videre omdannes til androgener.

Insulinniveauet virker desuden hæmmende på leverens produktion af SHBG. (6) SHBG har højere affinitet for testosteron end for østradiol, hvorfor den nedsatte mængde SHBG bevirker en øget mængde frit testosteron i blodet.

**PCOS og insulinresistens:**

**Insulinreceptoren:** Familiestudier har vist, at der er en øget risiko på 20 – 80% for udvikling af PCOS hos førstegradsslægtninge til patienter med PCOS (2,7,8,). Insulinreceptor genet fra PCOS patienter og fra raske er sekventeret med henblik på mutationer for at belyse om mutationer heri forårsager PCOS. PCOS patienter har kun ganske sjældent mutationer i insulinreceptor genet. (2,7-10). Muligvis skyldes insulinresistensen en insulin postreceptor defekt.

**Postreceptordefekter:** Fibroblaster fra PCOS patienter har uændret insulinreceptor mængde og affinitet sammenlignet med kontroller (11). Derimod ses ved PCOS en nedsat insulin postreceptor tyrosin phosphorylering og en øget serin phosphorylering. Dette forårsager en hæmning af tyrosin kinase aktiviteten i insulinreceptoren og dermed en nedsat effekt af insulin. Denne serin phosphorylering findes ikke hos patienter med type 2 diabetes (2,7,11). Hvorledes serin phosporyleringen forårsages er endnu ukendt (2,7,11).Et studie har vist, at serin phosphorylering af enzymet p450c17alfa kan øge dets aktivitet og dermed måske bidrage til hyperandrogenæmi (12).

Studier har vist, at mængden af GLUT-4 i adipocytters cellemembran er reduceret ved PCOS uafhængig af BMI (14). Den reducerede GLUT-4 mængde kan være et resultat af en insulin postreceptor defekt, som bremser postreceptorkaskaden og betinger en reduceret produktion af GLUT4.

**PCOS og risiko for type 2 diabetes:** For at undgå udvikling af diabetes ved udtalt insulinresistens er en velfungerende endogen insulinsekretion nødvendig. I et studie udført af Dunaif et al (15) fandt man at patienter med PCOS trods normal OGTT havde reduceret beta-celle respons sammenlignet med normale. Dette sås både hos normalvægtige og hos overvægtige patienter. Dette kan øge risikoen for udvikling af diabetes ved PCOS. Insulinresistensen kan udvikle sig til regulær type 2 diabetes ved insufficient evne til beta-celle respons. Insulinresistensen er til stede også hos normalvægtige med PCOS, men er mere udtalt hos overvægtige patienter (14,15).

**Gonadotropinsekretion ved PCOS:** Forstyrrelser i sekretionen af gonadotropiner er velbeskrevet ved PCOS. Således ses ved PCOS en øget frekvens og amplitude af LH sekretionen. Denne øgede LH sekretion skyldes sandsynligvis en hypothalamisk defekt, som øger sekretionen af GnRH, og dermed af LH. Tidligere studier har fundet, at denne hypothalamiske defekt kan opstå som følge af ændringer i det hormonelle miljø, for eksempel hyperandrogenæmi (16). Øget LH/FSH ratio benyttes ikke som kriterium ved diagnostikken af PCOS pga de pulsatile svingninger i disse hormoner.

**Cortisol og PCOS:** Tidligere studier har fundet forstyrrelser i hypothalamus-hypofyse-binyre aksen i form af øget cortisol sekretion ved PCOS. Denne tendens er øget hos overvægtige PCOS patienter (17-19). Binyrernes cortisol og androgenproduktion er øget trods uændret ACTH koncentration, hvilket danner baggrund for en hypotese om, at der ved PCOS ses en hyperreaktivitet af binyrerne med øget cortisol og androgen respons til følge (19).

**Væksthormonsekretion ved PCOS:** Insulinmængden og sekretionen af væksthormon hænger tæt sammen. I et studie af Morales et al (20) fandt man at normalvægtige patienter med PCOS havde samme GH pulsfrekvens som kontroller, men havde en 30 % øget pulsamplitude for GH. I modsætning hertil havde overvægtige PCOS patienter en svært nedsat GH sekretion pga nedsat pulsamplitude. Man konkluderede at de overvægtige patienter havde en øget mængde GHBP og en reduceret mængde IGFBP-1 som følge af insulins påvirkning af leveren. Den reducerede mængde IGFBP-1 bevirker en øget mængde IGF-1, som kan have effekt på ovariets sekretion af androgener. (20)

**Glitazoners effekt på insulinresistens**: Da hyperinsulinæmi tilsyneladende har essentiel betydning for udvikling af hyperandogenæmi ved PCOS, er der de sidste år lavet interventionsstudier, hvor man har forsøgt behandling af PCOS via reduktion af insulinresistens. Mange studier har benyttet metformin, men efter indregistrering af glitazonpræparaterne, er disse også undersøgt i enkelte studier. Man har dog kun undersøgt det nu afregistrerede troglitazon. I undersøgelserne har man fundet, at behandling med troglitazon medførte en øget insulinfølsomhed, en nedsat androgenkoncentration og en øget ovulationsrate hos patienterne (21-23). Den positive effekt på hormoner og metabolisme er dosisafhængig. Behandlingsvarigheden i studierne har varieret fra 12 – 44 uger. Kun i et enkelt af de nævnte studier indgik en placebogruppe (23). Man har ikke tidligere undersøgt, hvordan hormonprofilerne af væksthormon, cortisol og LH ændres under behandlingen med glitazoner. En eventuel ændring af muskel glukosemetabolisme og gen ekspression efter behandling med glitazoner er ikke undersøgt i de omtalte studier.

**Fordele og risici forbundet ved forsøget, etiske overvejelser:**

**Thiazolidindioner:**

Thiazolidindioner eller glitazon præparater er en relativt ny behandling til patienter med type 2 diabetes. Aktuelt benyttes disse præparater primært i kombinationsterapi med sulfonylurinstof eller metformin, hvor man ikke har kunnet opnå sufficient glykæmisk kontrol ved behandling med disse præparater alene. Glitazonerne er dog godkendt til monoterapeutisk behandling i USA.

Glitazonerne virker som agonister for den nukleare peroxisome-proliferator-aktiveret receptor-γ (PPAR-γ). Denne receptor findes i fedtvæv, skeletmuskulatur og lever. Via påvirkning af denne receptor reduceres insulinresistensen i fedtvæv, skeletmuskulatur og lever. Den eksakte virkningsmekanisme er dog ukendt. Reduktion i blodglukose indtræder relativt langsomt og er maksimal efter cirka 8 uger (24). Herhjemme er to glitazonpræparater indregistreret; rosiglitazon (Avandia, Glaxo Smithkline Pharma A/S) og pioglitazon (Actos, Ely Lilly Danmark A/S).

I dette studie vil patienterne blive behandlet med pioglitazon, tablet Actos à 30 mg, 1 tablet daglig.

**Farmakokinetik:** Fælles for de to glitazonpræparater er en hurtig og næsten fuldstændig optagelse fra mavetarmkanalen efter peroralt indtag. Biotilgængeligheden er cirka 80% for pioglitazon. Proteinbindingen i plasma er 99%. Pioglitazon metaboliseres i leveren, fortrinsvis via CYP2C8, disse metabolitter udskilles i urin og fæces.

Pioglitazon omdannes til delvist aktive metabolitter i leveren. Maksimal plasmakoncentration ses efter cirka 2 timer. Halveringstiden i plasma er 5-6 timer for pioglitazon, 16-23 timer for de delvist aktive metabolitter.

**Bivirkninger og forsigtighedsregler:** Tidligere er leverskade observeret ved anvendelse af et analogt præparat (troglitazon), hvorfor glitazonerne er kontraindicerede ved aktiv leversygdom. Der er dog ikke blevet gjort tilsvarende observationer ved anvendelse af pioglitazon eller rosiglitazon. I dette studie kontrolleres leverparametre løbende hos de indgående patienter, og patienter med forhøjede leverparametre ekskluderes.

Glitazoner kan hos enkelte give anledning til væskeretention specielt i form af lette ankelødemer. Muligvis på baggrund af dette har man observeret et ikke klinisk betydende fald i hæmoglobin koncentrationen efter behandling med glitazon præparater.

Glitazoner kan hos diabetespatienter forårsage en mindre vægtstigning.Ved behandling af patienter med PCOS har vægtøgningen i gennemsnit været på 1 kg. I tidligere udførte studier på PCOS patienter (21-23) er ingen patienter ekskluderet på grund af bivirkninger. Bivirkningsfrekvensen i et af studierne (23) er rapporteret til 4-7 %, mens der i de øvrige studier ikke er fundet bivirkninger til behandlingen.

Erfaringen med graviditet er begrænset, hvorfor patienterne får foretaget graviditetstest før inklusion i studiet og påbydes antikonception med kondom under studiet.

Personer med hjerteinsufficiens bør ikke behandles med glitazonpræparter. Patienter med hjertelidelse ekskluderes derfor fra undersøgelsen. Da risikoen for hjerteinsufficiens i denne aldersgruppe er lille, udføres ikke rutinemæssig EKKO kardiografi.

**Etiske aspekter:**

Som anført i indledningen er PCOS et syndrom, som på længere sigt kan bevirke udvikling af type 2 diabetes og iskæmisk hjertelidelse på baggrund af den tilstedeværende insulinresistens. Man har i dag ikke en behandlingsmetode, som kan reducere insulinresistensen, som er den udløsende årsag i syndromet. I stedet benyttes i dag oftest p-piller, som begrænser de kosmetiske gener ved PCOS og gør menstruationerne regelmæssige. P-piller bedrer dog ikke insulinfølsomheden og kan naturligvis ikke benyttes som behandlingsmetode ved graviditetsønske. Der er således behov for nærmere undersøgelser af behandlingsmetoder, som kan reducere insulinresistensen og øge fertiliteten hos disse patienter.

Da den muligt teratogene effekt af glitazoner ikke kendes, bør forsøgsdeltagerne undgå graviditet i behandlingsperioden. Når glitazonbehandling benyttes til behandling af diabetespatienter benyttes oftest p-piller som præventionsmetode. Dette er ikke muligt i ovennævnte forsøg, idet p-piller hæmmer produktionen af androgener i blodet. P-piller vil påvirke hormonkoncentrationerne i blodet så kraftigt, at effekten af Actos behandlingen sandsynligvis vil sløres. Idet forsøget kun løber over 16 uger finder vi, at det er urealistisk at alle 30 deltagere accepterer anlæggelse af spiral. I amerikanske forsøg har man i stedet accepteret brug af kondom eller pessar hos patienter i glitazonbehandling eller graviditetstest er blevet udført ved forsøgets begyndelse (21-23).

Vi vil i samtykkeerklæringen bede patienterne skrive under på, at de vil undgå graviditet under forsøgsperioden via kondom eller anden prævention uden hormonindhold. For at minimere risikoen for graviditet skal der benyttes dobbeltbarrieremetode; dvs kondom + pessar, kondom + sæddræbende creme eller pessar + sæddræbende creme. Det bliver i informationen ligeledes understreget, at Actos er et potentielt fosterskadeligt præparat, hvorfor graviditet skal undgås. Denne protokol har været indsendt til Etisk Komite for Fyn og Vejle Amt. Etisk komite har godtaget denne information. Actos’ danske repræsentant, Eli Lilly har ligeledes godkendt brugen af præparatet i forsøget.

Sammenholdes risikoen som følge af PCOS med den bivirkningsrisiko, som er stede ved anvendelse af pioglitazon, finder forskergruppen at gennemførelse af forsøget er etisk forsvarligt.

Alle præparater der indgår i undersøgelsen er markedsførte i Danmark. Undersøgelsen overholder Helsinki II deklarationen og anmeldes til Videnskabsetisk Komite og til Lægemiddelstyrelsen.

Alle patienterne informeres mundtligt og skriftligt. Det vil blive understreget, at deltagelse er frivillig og at samtykket til enhver tid kan trækkes tilbage, og at dette ikke vil få betydning for den videre behandling. Informationerne angående forsøget vil følge de nye retningslinjer vedrørende krav til information og samtykke per 1. december 2000. Alle medvirkende vil få udleveret skriftligt informationsmateriale, der henvises til dette bilag.

Vedrørende mundtlig information vil denne indbefatte en uddybning af den skriftlige information og en nærmere beskrivelse af forsøget.

**Dosering og behandlingsperiode:**

Behandling med Actos a’ 30 mg er valgt ud fra tidligere udførte studier, hvor effekten af behandlingen med glitazoner er dosisafhængig. Man har derimod ikke observeret øget bivirknings frekvens ved dosisøgning. Derfor har forskergruppen valgt maksimal dosering af præparatet. Da effekten af glitazoner først forventes at sætte ind efter 8 uger er der valgt en længere behandlingsperiode for at sikre maksimal effekt.

**Forsøgspopulation:**

Forsøgspopulationen består af 30 patienter med polycystisk ovariesyndrom. Patienterne rekrutteres via vores ambulatorium ud fra kriterier som anført under relevante afsnit.

**Formål:**

Formålet med studiet er følgende:

- At undersøge sammenhængen mellem insulin sekretion og insulinsensitivitet og glukoseudnyttelse hos normoglykæmiske, hyperinsulinæmiske kvinder med PCOS.
- At undersøge hvorvidt en reduktion af insulinresistensen under behandling med pioglitazon vil reducere androgenniveauet hos patienterne med PCOS.
- At klargøre mekanismerne hvorved pioglitazon påvirker glukose metabolismen og insulin effekten via måling af den intracellulære glukosemetabolisme i skeletmuskulatur før og efter behandling med pioglitazon hos PCOS patienter.
- At undersøge hvorledes ekspressionen af gener i muskelvæv hos PCOS patienter ændres efter behandling med pioglitazon via CHIP-array analyse.
- At klargøre hvorledes hypofyse-væksthormon aksen ændres under behandling med pioglitazon. Dette undersøges før og efter behandlingsperioden via basalmåling (døgnprofil for GH) og via stimulation (PD-GHRH-test).
- At klargøre hvorledes hormonprofilen af cortisol ændres under behandling med pioglitazon. Dette undersøges før og efter behandlingsperioden via døgnprofil for cortisol og via opsamling af urin-cortisol og metabolitter.
- At undersøge hvorledes niveauet af LH ændres under behandling med pioglitazon. Dette undersøges før og efter behandlingsperioden via LH døgnprofil måling.

**Design:**

**Endpoints:**

Primære endpoints: Glukoseinfusionshastigheden (M værdi) under euglykæmisk hyperinsulinæmik clamp.. Nærmere beskrevet under statistik afsnit.

Sekundære endpoints: BMI, LH, FSH, total og frit testosteron, faste blodsukker, faste c-peptid, FFA, areal under kurven (AUC) af insulin og BS ved OGTT, GH respons ved PD-GHRH test, glukoseomsætning ved euglykæmisk hyperinsulinæmisk clamp, FFA respons ved OGTT. Pulsatile og nonpulsatile svingninger af LH og cortisol ved døgnprofilmålinger, AUC for cortisol niveau ved døgnprofil.

**Forsøgstype/design:**

Dobbeltblindt placebokontrolleret randomiseret studie på 30 patienter med PCOS. Patienterne randomiseres til enten placebobehandling eller behandling med pioglitazon.

Patienter, som tidligere har været i ambulatoriet vil blive tilbudt deltagelse, hvis de opfylder inklusionskriterierne. Disse vil blive indkaldt til samtale via brev. Samtalen foregår i vores ambulatorium og patienten gøres opmærksom på muligheden for at medbringe en bisidder til denne samtale. Under samtalen gennemgås forsøget i henhold til vedlagte informationsmateriale og brochuren ”Før du beslutter dig” udleveres. Patienten tilbydes opfølgende samtale i ambulatoriet eller kontaktes per telefon en uge efter med henblik på stillingtagen til deltagelse. Såfremt patienten har behov for yderligere betænkningstid aftales dette individuelt, men forskergruppen finder 1 uges betænkningstid rimelig.

Der bliver ikke behov for at hente samtykke fra andre end forsøgspersonen selv, idet patienterne skal være myndige for at deltage i forsøget.

Hos patienterne med PCOS, som har uregelmæssige menstruationer med cykluslængde under 3 måneder afventes menstruation, hvorefter undersøgelser laves på cyklusdag 2 – 8. Hos patienter med PCOS og cykluslængde over 3 måneder laves undersøgelserne på et vilkårligt tidspunkt.

Før opstart med pioglitazon/ placebo laves følgende undersøgelser:

Dag 1 -2:

- **Objektiv undersøgelse**
- **Blodprøver**
- **Døgnprofil GH, LH, cortisol**
- **PD-GHRH test**
- **Dexa skanning**

Dag 3:

- **Euglykæmisk hyperinsulinæmisk clamp**
- **Muskelbiopsi (inklusiv CHIP og GLUT4 analyse)**

Ligeledes får alle patienter udført oral glukosetolerance test før opstart af behandlingsperioden for at sikre at patienterne ikke har diabetes. Denne undersøgelse udføres på et vilkårligt tidspunkt af menstruationscyklus og maksimalt være ½ år gammel.

Efter undersøgelserne opstartes behandling med tablet pioglitazon/ placebo i 16 uger. Under en af de sidste behandlingsdage udføres tilsvarende undersøgelser som ved inklusionstidspunktet på alle patienterne.

**Beskrivelse af de omtalte undersøgelser:**

**Objektiv undersøgelse:**

- Højde, vægt, BMI udregnes
- Talje-hofte mål
- Ferrimann-Gallway score
- BT, puls
- Stetoskopi af hjerte og lunger

**Laboratorieanalyser:**

- **Hormoner:** LH, FSH, Androgenstatus, østrogenstatus, prolaktin, leptin, IGF1, graviditetstest.
- **Metabolisme:** faste lipidstatus, faste BS, faste insulin, faste c-peptid, Hba1c, s-FFA
- **Andre:** Hgb, creatinin, elektrolytter, leverparametre
- **EKG**

Laboratorie analyser udføres på Klinisk kemisk afdeling, Odense Universitetshospital, Statens Seruminstitut og Rigshospitalet. Alle tests udføres ved Endokrinologisk afdeling M.

**Cortisol profil:**

- Måling af s-cortisol kl. 24, 1 ,2 ,3 ,4 ,5 ,6 ,7 og 8
- Opsamling af urin med henblik på måling af urin-cortisol og metabolitter.

**GH og LH døgnprofil:**

- Måling af GH og LH som anført nedenfor.

Udføres ved hjælp af IV pumpe:

Hepariniseret intravenøs kanyle (1,1x25mm Introducer, Carmeda, Sweden) placeres i underarmsvene. Blodudtagning starter efter min. 30 min. gennem et nontrombogent kateter (ConFlo System, Carmeda, Sweden) som forbindes til en peristaltisk pumpe (Swemed Lab Pump, Carmeda, Sweden). Herefter udtages blodprøver kontinuerligt. Flowhastighed 3 ml/t med skift til nyt glas med 20 min intervaller. Pumpen tillader at patienterne kan sove trods blodprøvetagning.

**PD-GHRH test:**

Foretages efter faste fra midnat. Testen er standardiseret til at begynde kl. 8.30.

- Til tid=0 indgives 120 mg pyridostigmin (mestinon, Hoffman La Roche, Switzerland)
- Til tid=60 min. Indgives GHRH, 1 g/ kilo legemsvægt ( Groliberin, Pharmacia or Geref, Serono, Italy).
- **Blodprøver:** væksthormon til tid 0, 20, 30, 45, 60, 90.

Bivirkninger til undersøgelsen er hos cirka 50% af patienterne rødme af ansigtshuden af cirka 30 sekunders varighed. Cirka 10% vil få oppustet mave eller tics/akkomodationsbesvær. Cirka 1% vil få diarre og i sjældne tilfælde kvalme og opkastninger.

**Dexa-skanning:**

- Lumbal, hofte og whole body
- Kropssammensætning

Skanningen varer ca. et kvarter og medfører ikke ubehag. Strålemængden ved undersøgelsen svarer til ca. 1/7 af baggrundsstrålingen per år i Danmark.

**Oral glukose tolerance test**

Foretages efter 10 timers faste.

- Peroral indtagelse af 75 g glukose til t=0.
- **Blodprøver**: Plasma glukose, c-peptid, s-FFA og insulin til tid -10, 0, 30, 60, 90, 120, 180 min.

**Euglykæmisk hyperinsulinæmisk clamp og kalorimetri**

Foretages efter 10 timers faste. Før opstart af undersøgelsen anlægges to velfungerende drop. Det ene drop benyttes til blodprøvetagning, det andet drop benyttes til infusion af insulin og glukose. Den arm, hvorfra der udtages blodprøver placeres i varmekasse.

- Initialt startes infusion af tritieret glukose (primed, surface adjusted) til bestemmelse af den hepatiske glukoseproduktion.
- Efter 1½ times basal periode tilkobles patienten i ½ time det indirekte kalorimeter til bestemmelse af glukose- og lipidoxidationen. Efter den indirekte kalorimetri udføres **muskelbiopsi** i m. vastus lateralis vha. Bergstrøm-kanyle.
- Herefter startes en kontinuerlig infusion af insulin (40 mU/min/m^2^). Plasma glukosekoncentrationen fastholdes (clampes) på 5 mM ved en variabel infusion af 18 % glukose (tilsat tritieret glukose; HOT-GINF) i alt 3 timer. Glukose infusions hastigheden (GINF) i den sidste ½ time benyttes til beregning af patientens insulinfølsomhed.
- I den sidste ½ time udføres atter indirekte kalorimetri og patienten får atter udført en muskelbiopsi.
- **Blodprøver:** I løbet af undersøgelsen tages blodprøver fra til bestemmelse af plasma glukosekoncentrationen samt til bestemmelse af insulin, C-peptid, FFA, og tritieret glukosekoncentration. I alt fratages ca. 200 ml blod

**Muskelbiopsi:**

- Muskelbiopsi udføres 2 gange under hver clamp, begge gange umiddelbart efter kalorimetrimålingen.
- Anlæggelse af lokalbedøvelse i underhuden på lårets yderside.
- Efter cirka 10-20 minutter udtages muskelbiopsien med en muskelbiopsikanyle. I alt udtages cirka 200 mg muskelprøve.

Muskelbiopsierne nedsænkes øjeblikkelig efter udtagelsen i flydende kvælstof og bliver efterfølgende undersøgt for nøgleenzymer og substrater involveret i glukosemetabolismen og i insulin-signal-kaskadereaktionen, ligesom muskelindholdet af triglycerid vil blive undersøgt og muskelens fibersammensætning vil blive bestemt.

**CHIP-array undersøgelse:** Udføres på oprensede muskelceller.

**Randomisering/blinding:** Monitor sørger for nummerering af medicin. Glas nummereres fortløbende fra 1-30, således at der er tilfældig fordeling af placebo og aktiv medicin. Glassene udleveres til patienterne fortløbende således at den først inkluderede får udleveret glas 1.

Blindingen sikres ved at kun monitor kender randomiseringskoderne og at glassene er mærket enslydende uanset indholdsstof.

**Forsøgsbehandling:** Placebotabletter og det aktive præparat omhældes på glas via Centralapoteket, OUH. Glassene indeholder tabletter svarende til hele behandlingsperioden. Patienterne indtager 1 tablet daglig ved morgenmåltidet.

Vedrørende etikettering: Mærkningen af glassene er som følger: Til klinisk forsøg. Dosering: 1 tablet daglig. 140 tabletter. Kodenummer fra 1-30. Batchnummer. Udløbsdato. Endokrinologisk afdeling M, Odense Universitetshospital.

**Forsøgsperioder:** Behandlingsperioden for den enkelte deltager er 16 uger, eventuelt 4 uger længere idet forsøgsdeltagerne så vidt som muligt undersøges i follikulærfasen af menstruationscyklus som anført ovenfor. Der er ikke tale om nogen run-in periode idet patienterne undersøges før opstart af behandling og umiddelbart før seponering.

**Afbrydning af behandling:** Behandlingen af den enkelte patient kan afbrydes såfremt den pågældende patient får uacceptable bivirkninger eller såfremt der er mistanke om non-compliance.

Af uacceptable bivirkninger er en fordobling af leverparametre i forhold til udgangsværdi eller bivirkninger som er så subjektivt generende for den enkelte patient, at denne ikke ønsker at fortsætte behandlingen. Herunder medregnes vægtstigning.

Der bliver ikke tale om at investigator afbryder forsøget idet der er tale om en relativt kort behandlingsperiode.

**Kontrolprocedurer:** Indtagelse af medicin kontrolleres via tablettælling ved midten af behandlingsperioden og ved afslutning.

**Randomiseringskoder:** Randomiseringskoden opbevares af monitor. Kodebrud kan komme på tale ved mistanke om systematisk optræden af bivirkninger i den aktive behandlingsgruppe. Ligeledes kan der blive tale om kodebrud såfremt der optræder alvorlig sygdom hos en forsøgsdeltager, som nødvendiggør, at patientens medicinforbrug kendes.

**CRF-data:** På Case Report Forms rapporteres følgende data: Vægt, BT, puls, hgb, leukocytter, leverparametre, creatinin. Desuden registreres eventuel interkurrent sygdom og subjektive bivirkninger.

**Udvælgelse af forsøgspersoner:**

**Inklusionskriterier:**

Uregelmæssige menstruationer, dvs cykluslængde >36 dage

præmenopausal

Forhøjet faste insulin > 50 pmol/l.

Forhøjet frit testosteron > 0,035 nmol/l.

**Eksklusionskriterier**

Alder<18 år.

P-piller indenfor 3 måneder

Postmenopausal (forhøjet FSH)

Kendt diabetes mellitus

Endokrinologisk eller anden behandlingskrævende sygdom

Spise forstyrrelse eller kendt psykiatrisk anamnese

Medicinforbrug, som vides at påvirke parametre der undersøges i denne undersøgelse.

Graviditet

Planlagt graviditet i behandlingsperioden

Tidligere eller aktuel leverlidelse eller forhøjede leverparametre

Tidligere eller aktuel hjertelidelse

**Udgang af forsøget:** Efter 16 ugers behandling eller som anført ovenfor seponeres behandlingen.

Forsøgspersoner, som udgår pga bivirkninger til medicinen får tilbudt opfølgende samtale en måned efter udgang af forsøget eller i øvrigt ved behov. Under dette besøg foretages måling af CRF data og i øvrigt afhængig af patientens symptomer. Såfremt patienten er udgået på grund af manglende compliance tilbydes ligeledes opfølgende samtale med måling af CRF data.

**Behandling af forsøgspersoner:**

**Medicin:** Aktiv behandlingsgruppe: Tablet Actos á 30 mg. 1 tablet per os morgen.

Placebogruppe: Placebotablet. 1 tablet per os morgen.

**Ledsagende medicin:** Patienterne må ikke indtage hormonbehandling eller anden medicin som formodes at påvirke hormonkoncentrationer eller glukosemetabolisme. Patienterne kan indtage håndkøbsmedicin i begrænset mængde, men kun medicinprodukter, som ikke indvirker på ovenstående. Ligeledes frarådes naturmedicin.

**Compliance check:** Udføres ved hjælp af tablettælling.

**Efterfølgende behandling:** Patienterne kan efter eget ønske genoptage eventuel p-pille behandling eller anden tidligere behandling efter udgang af forsøget.

**Effektvurdering:**

**Sikkerhedsparametre:** Af sikkerhedsparametre benyttes ovenstående CRF parametre. Valget af disse parametre er sket ud fra tidligere udførte forsøg med glitazonbehandling i denne patientpopulation. I disse forsøg er ikke rapporteret bivirkninger ud over en svag tendens til vægtstigning. Øvrige effektparametre er valgt ud fra ovennævnte bivirkningsprofil ved behandling med glitzonpræparater hos diabetespatienter.

**Vurdering af sikkerhedsparametre:** Forsøgsdeltagerne følges en gang om måneden eller med kortere intervaller såfremt der er behov for dette. Ved disse ambulante besøg udføres klinisk undersøgelse samt blodprøvemonitorering som anført ovenfor.

**Bivirkningsrapportering:** Indberetning af bivirkninger til Lægemiddelstyrelsen sker jævnfør givne regler for dette.

**Komplikationsbehandling og opfølgning:** I henhold til de aktuelle erfaringer med glitazonbehandling forventes ikke komplikationer til behandlingen. Såfremt der skulle forekomme leverenzymstigning, faldende hæmoglobin eller større vægtstigning som kan tilskrives den medicinske behandling følges forsøgspersonen indtil dette har normaliseret sig. Forsøgskomplikationer imødegås i øvrigt ved at forsøgsdeltagerne møder til ovennævnte kontroller.

**Statistik:**

**Statistiske metoder:** Effekten af behandling undersøges ved uparret og parret t-test. Signifikans niveauet sættes til 5%. Til vurdering af pioglitazons effekt på nedenstående parametre benyttes således værdierne fra patienternes undersøgelsesresultater ved inklusion og ved afslutning af behandlingsperioden. Resultaterne fra mellemliggende kontroller indbefatter kun data vedrørende compliance og bivirkninger til behandlingen. Disse resultater kan ikke benyttes til vurdering af pioglitazonbehandlingen i øvrigt. Interim analyser optræder ikke.

Behandlingseffekten vurderes på følgende parametre: frit testosteron, faste insulin, total testosteron, faste blodsukker, faste c-peptid, FFA, areal under kurven af insulin og BS ved OGTT, GH respons ved PD-GHRH test, glukoseomsætning ved euglykæmisk hyperinsulinæmisk clamp, FFA respons ved OGTT, AUC for cortisol døgnprofil.

Desuden beregnes ændringer før og efter behandlingsperioden i pulsatile og nonpulsatile svingninger af GH og LH ved døgnprofilmålinger. Disse ændringer analyseres efter deconvolutionsanalyse som beskrevet ved Velthuis et al (25,26).

**Styrkeberegning.** Styrkeberegningen er foretaget ud fra reference 27. I dette studie er benyttet tilsvarende insulin infusion under clamp som i dette studie. Beregningen forudsætter en type 1 fejl på 0,05 og en type 2 fejl på 0,1. Standard deviationen for insulinfølsomhed under clamp sættes til 1,5 og vi ønsker at observere en forskel på minimum 2 mg/kg per min. Under disse forudsætninger kræves 12 patienter i hver gruppe. For at tage højde for eventuelt frafald inkluderes 15 patienter i hver gruppe.

**Kriterier for afslutning:** Forsøget afsluttes efter 16 ugers behandling eller som anført ovenfor.

**Manglende data:** Opgørelse af behandlingseffekt kræver at projektdeltagerne får udført undersøgelserne både ved inklusion og ved ophør af behandlingsperioden. Ønsker projektdeltageren ikke at få udført muskelbiopsi accepteres dette. Med henblik på compliance accepteres forglemmelse af max 10% af tabletterne.

**Forsøgspersoner i statistisk analyse:** I statistisk analyse vil indgå data fra de forsøgsdeltagere, som får udført parakliniske undersøgelser og tests i forbindelse med 16 ugers behandling med pioglitazon. Forsøgsdeltagere, som ikke fuldfører behandlingsperioden vil ikke indgå i beregningerne jævnfør ovenstående argumentation.

**Adgang til kildedata**

Kildedata indbefatter patientjournaler og laboratoriesvar. Investigator tillader direkte adgang til kildedata og dokumenter ved monitorering, auditering og inspektion fra etisk komite og Lægemiddelstyrelsen. Forsøgspersonerne accepterer dette i vedlagte fuldmagt. (bilag 4)

**Kvalitetssikring**

Det bekræftes at de almindelige procedurer for kvalitetskontrol og kvalitetssikring følges jfr. GCP guidelines afsnit 1.46 og 1.47.

**Håndtering og arkivering af data**

Forsøgsresultater opbevares sammen med CRF i separat chartek. Disse opbevares under og efter forsøget på afdeling M, Odense Universitetshospital i aflåst arkiv. Kun personer i investigator gruppen har adgang til materialet. Dataresultater overføres til database. Databasen valideres ved hjælp af dobbelt dataindtastning. Data opbevares ifølge anvisninger fra Datatilsynet.

**Finansiering og forsikring**

Forsøget tilstræbes finansieret via private fonde. Udgifter til lægeløn sker gennem ph.d stipendium.

Faciliteter til udførelse af tests haves i afdeling M, og det forventes at forsøget kan gennemføres uden ansættelse af nyt personale.

Forsikring: Deltagerne er indbefattede af de almindelige patientforsikringsregler.

**Retningslinjer for publikation**

Resultater fra projektet kommer til at indgå som en del af en ph.d afhandling og søges i øvrigt publiceret som:

Glintborg D, Henriksen JE, Andersen M, Hagen C, Hermann P: Effect of pioglitazone on glucose metabolism and insulin resistance in women with PCOS.

Glintborg D, Andersen M, Henriksen JE, Hagen C, Hermann P: Effect of pioglitazone on growth hormone, LH and cortisol in women with PCOS.

**Blodprøveanalyser:**

**Ved inklusion og afslutning:**

**Blodprøvaanalyser:**

Hormonanalyser: LH, FSH, androgenstatus, østrogenstatus, prolaktin, leptin, IGF1 - total: 18ml.

Metabolisme: lipider, insulin, c-peptid, Hba1c, FFA – total: 19 ml.

Andre: Hgb, creatinin, natrium, kalium, væsketal. – total: 7 ml.

**LH, GH profil:**

72 x LH, 72 x GH. – total: 252 ml.

**Cortisol profil:**

9 x cortisol. – total: 31,5 ml.

**Pd-GHRH test:**

9 x GH. – total: 42 ml.

**OGTT:**

7 x BS, 7 x c-peptid, 7 x FFA, 7 x insulin. - total: 84 ml.

**Hyperinsulinæmisk euglukæmisk clamp:**

Insulin, c-peptid, FFA, tritieret glukose. – total: 200 ml.

**Kontrolbesøg.**

Levertal, Hgb, Leu, natrium, kalium. – total: 7 ml.

#### Bilag

1. Lægmandsresume
2. Patientinformation
3. Fuldmagt vedrørende direkte adgang til kildedata og dokumenter.

Bilag 1: 29. august 2026

**Lægmandsresume: Undersøgelse vedrørende effekt af pioglitazonbehandling på kvinder med øget behåring.**

I undersøgelsen ønsker vi at belyse hvorledes behandling med pioglitazon påvirker kvinder med øget behåring.

Kvinder med øget behåring er ofte overvægtige og har øget mængde mandligt kønshormon i blodet. Deres blodprøver viser hyppigt forandringer, som minder om det, man ser hos patienter med sukkersyge.

Pioglitazon er er præparat, som benyttes til at øge følsomheden for insulin hos patienter med sukkersyge. Tidligere undersøgelser har vist, at dette præparat kan sænke niveauet af insulin i blodet hos kvinder med øget behåring. Ligeledes reduceres niveauet af mandlige kønshormoner under behandlingen. Præparatet har kun været markedsført i få år, og det er endnu ikke undersøgt hvorledes en række andre hormoner, herunder væksthormon og stresshormoner, ændres under behandlingen.

I denne kliniske undersøgelse skal indgå en gruppe på 30 kraftigt behårede kvinder. Patienterne randomiseres til placebo eller til det aktive præparat. Behandlingsperioden er i alt 16 uger.

For at indgå i forsøget skal man være rask og ikke tage medicin, som kan ændre undersøgelsesresultaterne. Herunder må personerne ikke indtage p-piller eller anden hormonbehandling.

I forbindelse med undersøgelsen udføres følgende på alle deltagere i projektet

- Sukkerbelastningstest med blodsukkermåling efter indtagelse af sukkervand.
- Klinisk undersøgelse, dvs højde og vægt samt vurdering af behåringsgrad.
- Blodprøver, som skal belyse hormonstatus.
- Måling af stress hormoner og overordnede kønshormoner gennem et døgn.
- Stimulationstest med måling af væksthormon.
- Undersøgelse, hvor patienten får målt følsomhed for insulin (clamp). Under denne undersøgelse udtages muskelprøver til måling af enzymer og DNA produktion.
- Knogleskanning, som måler knoglernes styrke og fordelingen af fedt og muskel.

Dette undersøgelsesprogram udføres før opstart af behandling med pioglitazon eller placebo og efter 16 uger. Undersøgelserne kræver 2½ dages indlæggelse.

Formålet med undersøgelsen er at få mere viden om, hvorfor nogle kvinder får øget behåring.

Vi ønsker at belyse om behandling med pioglitazon kan reducere mængden at mandligt kønshormon i blodet. Desuden vil vi gerne undersøge, hvordan niveauet af stresshormon og væksthormon ændres, når mængden af insulin i blodet reduceres.

Alle medvirkende får skriftlig og mundtlig information om ovenstående og kan afstå fra at medvirke, uden at dette får konsekvenser for videre udredning eller behandling.

Bilag 2 29. august 2026

**Patient information**

Du er henvist til afdeling M på grund af gener fra øget behåring. For at finde årsagen til dine gener har du gennemgået et rutine undersøgelses program, som blandt andet har undersøgt æggestokke og binyrer.

Man har gennem de senere år fundet, at mange kvinder med behåringsgener har en øget risiko for at udvikle sukkersyge. Du har derfor fået lavet en sukkerbelastnings undersøgelse, som skal belyse om du er en af de kvinder, som kan udvikle sukkersyge på længere sigt. Din undersøgelse har vist, at insulinniveauet i dit blod ligger over gennemsnittet. Muligvis kan du derfor have gavn af en type sukkersygemedicin, som reducerer insulin niveauet i blodet. Hvis insulinkoncentrationen reduceres vil risikoen for af udvikle sukkersyge mindskes. Undersøgelser har vist, at hvis mængden af insulin i blodet reduceres, vil niveauet af mandlige kønshormoner i blodet også reduceres.

Vi vil derfor spørge dig, om du vil deltage i en videnskabelig undersøgelse, som skal belyse dette nærmere.

Deltagelse i undersøgelsen indbefatter behandling med Actos (pioglitazon) i 4 måneder. Actos er et relativt nyt præparat i Danmark, som benyttes til behandling af patienter med sukkersyge. Formålet med undersøgelsen er at vise, hvordan dette stof påvirker hormoner og insulin hos kvinder med øget behåring. Hvis du accepterer at deltage i undersøgelsen vil du deltage i en lodtrækning, som afgør om du skal behandles med Actos eller med en virkningsløs tablet (placebo). Behandling med placebo er nødvendig, da der også kan være en ændring af hormonerne uden behandling. Hverken du eller lægerne vil vide, om du behandles med placebo eller Actos, før de 4 måneder er gået.

I undersøgelsen deltager i alt 30 patienter, hvoraf halvdelen vil modtage Actos behandling og halvdelen placebo.

Deltagelse i forsøget indbefatter, at du får lavet følgende undersøgelse før behandlingsperioden:

- Sukkerbelastningstest med indtagelse af et glas sukkervand og udtagning af blodprøver i alt 7 gange i løbet af 3 timer. Testen forgår efter 8 timers faste. Der er ingen bivirkninger til denne undersøgelse.

Før og efter behandlingsperioden får du lavet følgende undersøgelser:

- Blodprøver og klinisk undersøgelse.
- Indlæggelse et døgn til måling af hormonkoncentrationer i blodet og i urinen. Under undersøgelsen tages der løbende blodprøver fra et drop i den ene arm. Udover dropanlæggelse er der ingen bivirkninger.
- Hormontest med stimulation af væksthormondannelse og måling af hormonkoncentration. Dannelsen af væksthormon stimuleres ved indtagelse af en tablet og efter en time indsprøjtning af et stimulerende hormon. I løbet af undersøgelsen tages blodprøver for at måle væksthormondannelsen. Bivirkninger til denne undersøgelse er hyppigst rødme af ansigtshuden af cirka 30 sekunders varighed. Sjældnere opleves oppustet mave eller forbigående tics/uklart syn. Hos cirka 1% ses diarre, kvalme og opkastninger.
- Knogleskanning, som belyser knoglernes styrke og kroppens sammensætning af muskler og fedt. Strålemængden ved denne undersøgelse svarer til 1/7 af baggrundsstrålingen per år i Danmark.
- Udvidet undersøgelse af insulins virkning på sukker omsætningen i blodet. Denne undersøgelse varer cirka 8 timer og kræver ligeledes at du er fastende. Undersøgelsen foregår ved, at der lægges 2 drop, det ene benyttes til indsprøjtning af sukkervand og insulin, det andet til blodprøvetagning. Den arm, som benyttes til blodprøvetagning skal under undersøgelsen ligge i en varmekasse. Under undersøgelsen udtages to muskelprøver fra det ene lår. Muskelprøverne udtages med en nål i lokalbedøvelse. Udtagningen af muskelprøve kan være forbundet med ubehag trods bedøvelse og i sjældne tilfælde kan der optræde komplikationer i form af blodansamling eller infektion. Enkelte patienter kan have fornemmelsen af at have fået et ”trælår” i et par dage efter undersøgelsen. I løbet af undersøgelsen udtages i alt 200 ml blod til analyse.

Undersøgelsesprogrammet kræver 2½ dages fremmøde før og efter behandlingsperioden.

Under behandlingsperioden vil du blive fulgt løbende i ambulatoriet. Hvis der er mistanke om bivirkninger til behandlingen og du ikke kan tåle medicinen, kan denne naturligvis ophøre. De hyppigste bivirkninger til Actos er væskeophobning i kroppen. I gennemsnit tager man 1-2 kg på, men du taber dette igen efter ophør med behandlingen. Nogle patienter kan også få let mavebesvær.

Man har kun begrænset erfaring med Actos behandling og graviditet, men man har mistanke om, at stoffet kan være fosterskadeligt. Du vil derfor blive bedt om at skrive under på, at du vil benytte kondom/ pessar kombineret med sæddræbende creme i behandlingsperioden for at undgå graviditet. Da p-piller og andre præventionsmidler, som indeholder hormoner påvirker analyseresultaterne kan disse ikke benyttes i undersøgelsesperioden.

Denne deltagerinformation vedlægges informationsskrivelsen ”Før du beslutter dig”. Her kan du læse mere om dine rettigheder ved deltagelse i en videnskabelig undersøgelse.

Du har ret til betænkningstid før du accepterer at medvirke, og hvis du ønsker det kan du medbringe en bisidder til den mundtlige information.

Forsøget er omfattet af tavshedspligt vedrørende dine helbredsforhold, private forhold og andre fortrolige oplysninger. Du har mulighed for at få aktindsigt, hvis du ønsker dette. Hvis der skulle opstå gener som følge af forsøget, har du ret til at klage over dette.

Til forsøget søges i alt 850.000 kr. i støtte fra private fonde.

Deltagelse i denne undersøgelse er naturligvis ganske frivillig, og du kan til enhver tid trække dit samtykke tilbage uden at dette vil få nogen konsekvenser for den aktuelle eller videre behandling af dig.

Undersøgelsen er godkendt af den lokale videnskabsetiske komite og af Lægemiddelstyrelsen.

Undertegnede er informeret om undersøgelsen skriftligt og mundtligt:

Odense d. Navn:



Ligeledes skriver jeg under på, at jeg i behandlingsperioden vil undgå graviditet ved at bruge kondom eller anden prævention uden hormonindhold kombineret med sæddræbende creme.



Ansvarlig forskers attest for udlevering af skriftligt informationsmateriale og afgivelse af mundtlig information.

Odense d.



Venlig hilsen

Dorte Glintborg, Jan Erik Henriksen, Marianne Andersen,

Reservelæge 1. reservelæge, ph.d 1. reservelæge, ph.d

Pernille Hermann, Claus Hagen

1.reservelæge, ph.d overlæge, dr.med

Endokrinologisk afdeling M

Odense Universitetshospital

Kløvervænget 6, 4. sal.

5000 Odense C

65411810

Bilag 3: 29. august 2026

NAVN

ADRESSE

MM

CPR. NR:

Odense XXYYZZ

Kære navn

Som led i din deltagelse i undersøgelsen af insulinniveauets betydning for udvikling af uønsket behåring ved afdeling M, Odense Universitets Hospital, er der af myndighederne fastsat nye retningslinier for opbevaring og kontrol af de oplysninger, vi indsamler.

ASom led i alle lægevidenskabelige undersøgelser er det nu påkrævet, at en uvildig instans har adgang til at kontrollere, at undersøgelsen følger den gældende lovgivning.@ Dette gælder derfor også den undersøgelse, du deltager i.

Den uvildige instans er den såkaldte GCP enhed (enhed for Good Clinical Practice), der er nedsat af Fyns Amt. GCP-enheden kontrollerer videnskabelige undersøgelser på vegne af Videnskabsetisk Komite og Lægemiddelstyrelsen.

Vi skal derfor i henhold til loven bede dig give tilladelse til, at GCP enheden eller anden repræsentant for de Videnskabsetiske Komiteer eller Lægemiddelstyrelsen må kontrollere de oplysninger, vi indsamler om dig. Oplysningerne indbefatter journal og blodprøvesvar.

Din fuldmagt gælder i 15 år, men kan til enhver tid trækkes tilbage.

**Jeg giver hermed min fuldmagt til, at oplysninger om mig, der indsamles i forbindelse med undersøgelsen af insulins betydning for udvikling af uønsket behåring kan blive gennemgået af medarbejdere fra GCP enheden, Lægemiddelstyrelsen eller de Videnskabsetiske Komiteer i forbindelse med kontrol af data. Oplysningerne forbliver på Endokrinologisk afdeling M, Odense Universitets Hospital og videregives ikke til andre.**

**Denne fuldmagt indhentes i henhold til ' 20 i ALov om patienters retsstilling@.**

**Fuldmagten gælder KUN oplysninger, der har relation til projektet, og den kan til enhver tid tilbagekaldes.**

___________________ _________________________________

Dato Underskrift

**Litteraturhenvisninger.**

- 1. Franks S (1995) Polycystic ovary syndrome. New England Journal of Med. 333: 853-861.
  2. Dunaif A (1997) Insulin resistance and the polycystic ovary syndrome. Mechanism and implications for pathogenesis. Endocrine reviews 18(6): 774-800.
  3. Nestler JE, Jakubowicz DJ (1996) Decreases in ovarian cytocrome p450c17alfa activity and serum free testosterone after reduction of insulin secretion in polycystic ovary syndrome. New England Journal of Med. 335:617-23.
  4. Ünlühizarca K, Kelestimur F, Bayram F et al (1999) The effects of metfomin on insulin resistance and ovarian steroidogenesis in women with the polycystic ovary syndrome. Clinical Endocrinology 51: 231-236.
  5. La Marca A, Egbe TO, Morgante G et al (2000) Metformin treatment reduces ovarian cytochrome p450c17alfa response to human chorionic ganadotropin in women with insulin resistance related polycystic ovary syndrome. Hum Reprod 15:21-23.
  6. Nestler JE, Powers LP, Matt DW (1991) A direct effect of hyperinsulimemia on serum sex hormone-binding globulin levels in obese women with the polycystic ovary syndrome. J Clin Endocrinol Metab. 72:83-89.
  7. Legro RS, Spielman R, Urbanek M, et al (1998) Phenotype and genotype in polycystic ovary syndrome. Recent progr in hormone research 53: 217-256.
  8. Ciampelli M, Lanzone A (1998) Insulin and polycystic ovary syndrome: a new look at an old subject. Gynecol Endocrinol 12:277-292.
  9. Talbot JA, Bicknell EJ, Rajkhowa M, Krook A, et al (1996) Molecular scanning af the insulin receptor gene in women with polycystic ovary syndrome. J Clin Endocrinol Metab 81:1979-1983.
  10. Urbanek M, Legro RS, Driscoll D, Strauss J, et al (2000) Searching for the polycystic ovary syndrome genes. J Pediatr Endocrinol Metab 13:1311-1313.
  11. Dunaif A, Xia J, Book C, Schenker E, Tang Z. (1995) Excessive insulin receptor serine phoshorylation in cultured fibroblasts and in skeletal muscle: a potential mechanism for insulin resistance in the polycystic ovary syndrome. J Clin Invest 96:801-810.
  12. Zhang L, Rodriguez H, Ohno S, Miller WL. (1995) Serine phosphorylation of human P450c17 increases 17, 20 lyase activity: implications for adrenarche and for the polycystic ovary syndrome. Pcoc Natl Acad Sci USA. 92:10619-10623.
  13. Dunaif A, Segal KR, Shelley DR, Green G, Dobrjansky A, et al. (1992) Evidence for distinctive and intrinsic defects in insulin action in polycystic ovary syndrome. Diabetes 41:1257-1266.
  14. Rosenbaum D, Haber RS, Dunaif A. (1993) Insulin resistance in polycystic ovary syndrome: decreased expression of GLUT-4 glucose transporters in adipocytes. Am J Physiol 264(Endocrinol Metab 27) 197-202.
  15. Dunaif A, Finegood DT. (1996) B-cell dysfunction independent of obesity and glucose intolerance in the polycystic ovary syndrome. J Clin Endocrinol Metab 81:942-947.
  16. Dunaif A, Thomas A. (2001) Current concepts in the polycystic ovary syndrome. Annu rev med 52:401-9.
  17. Invitti C, De Maritn M, Delitala G, Velhhuis JD et al.(1998) Altered morning and nighttime pusatile corticotropin and cortisol release in polycystic ovary syndrome. Metabolism Feb;47(2):143-8.
  18. Miller JE, Bray MA, Faiman C, Reyes FI. (1994) Characterisation of 24-h cortisol release in obese and nonobese hyperandrogenic women. Gynecol Endocrinol Dec;8(4):247-54.
  19. Azziz R, Black V, Hines A, Fox LM, Boots LR. (1998) Adrenal androgen excess in the polycystic ovary syndrome: sensitivity and responsivity af the hypothalamic-pituritary-adrenal axis. J Clin Endocrinol Metab 83:2317-2323.
  20. Morales AJ, Laughlin GA, Bützow T, Maheshwari H, Baumann G, Yen SSC. (1996) Insulin, somatotropic, and luteinizing hormone axes in lean and obese women with polycystic ovary syndrome: common and distinct features. J Clin Endocrinol metab 81:2854-2864.
  21. Dunaif A, Scott D, Finegood D, Quintana B, Whitcomb R. (1996) The insulin-sensitizing agent troglitazone improves metabolic and reproductive abnormalities in the polycystic ovary syndrome. J Clin Endocrinol Metab 81:3299-3306.
  22. Ehrmann DA, Schneider DJ, Sobel BE, Cavaghan MK, Imperial J, Rosenfield RL, Polonsky KS. (1997) Troglitazone improves defects in insulin action, insulin secretion, ovarian steroidgenesis, and fibrinolysis in women weith polycystic ovary syndrome. J Clin Endocrinol Metab 82:2108-2116).
  23. Azziz R, Ehrmann D, Legro RS, Whitcomb RW, Hanley R, et al. (2001) Troglitazone improves ovulation and hirsutism in the polycystic ovary syndrome: A multicenter, double blind, placebocontrolled trial. J Clin Endocrinol Metab 86:1626-1632.
  24. Schmitz OE, Brock B, Madsbad S, Beck-Nielsen H. (2001) Thiazolidinedioner –en ny klasse af orale antidiabetika. Ugeskr laeger 163:6106-6110.
  25. Velthuis JD, Carlson ML, Johnsom ML (1987). The pituritary gland secretes in bursts: appraising the nature of glandular secretoty impulses by simultaneous multiple-parameter deconvolution of plasmahormone concentrations. Proc Natl Acad Sci USA 84:7686-7690.
  26. Veldhuis JD, Faria A, Vance ML, Evans WS, Thorner MO, Johnson ML (1988). Contemporary tools for the analysis of episodic growth hormone secretion and clearance in vivo. Acta Peadiatr Scand Suppl. 347:63-82.
  27. Diamanti-Kandarakis E, Kouli C, Tsianateli T, Bergiele A (1998). Theurapeutic effects of metformin on insulin resistance and Hyperandrogenism in polycystic ovary syndrome. European J Endocrinol 138: 269-274.
